# KRAS mutations promote the intratumoral colonization of enterotoxigenic bacteroides fragilis in colorectal cancer through the regulation of the miRNA3655/SURF6/IRF7/IFNβ axis

**DOI:** 10.1080/19490976.2024.2423043

**Published:** 2024-11-10

**Authors:** Yizhen Chen, Shaolin Liu, Song Tan, Yuanyuan Zheng, Yifan Chen, Changshun Yang, Shengtao Lin, Yulong Mi, Weihua Li

**Affiliations:** a Shengli Clinical Medical College of Fujian Medical University, Fuzhou, China; b Department of Gastrointestinal Surgery, Fujian Provincial Hospital, Fuzhou University Affiliated Provincial Hospital, School of Medicine, Fuzhou University, Fuzhou, Fujian, China; c Department of Geriatric Medicine, Fujian Key Laboratory of Geriatrics Diseases, Fujian Provincial Center for Geriatrics, Fujian Provincial Hospital, Fuzhou University Affiliated Provincial Hospital, School of Medicine, Fuzhou University, Fuzhou, Fujian, China

**Keywords:** Colorectal cancer, KRAS, tumor microenvironment, intratumoural microbiota, enterotoxigenic bacteroides fragilis, colonization

## Abstract

KRAS mutations are associated with poor prognosis in colorectal cancer (CRC). Although the association between the gut microbiota and CRC has been extensively documented, it is unclear whether KRAS mutations can regulate the gut microbiota. Metagenomics has identified changes in the diversity of the gut microbiota in CRC due to KRAS mutations. Specifically, KRAS mutations positively correlate with the abundance of the bacteroides. Understanding how to regulate the classic carcinogenic bacterium within the bacteroides, such as enterotoxigenic bacteroides fragilis (ETBF), to enhance treatment efficacy of tumors is a key focus of research. Mechanistically, we found that the reduction of miR3655 is indispensable for KRAS mutation-promoted proliferation of CRC and the abundance of ETBF. miR3655 targets SURF6 to inhibit its transcription. Further transcriptomic sequencing revealed that SURF6 promotes intratumoral colonization of ETBF in CRC by inhibiting the nuclear translocation and transcription levels of the IRF7, affecting the activation of the IFNβ promoter. Regulating miR3655 and SURF6 can promote IFNβ secretion in CRC, directly killing ETBF. These data indicate that KRAS mutations affect the intratumoral colonization of ETBF in CRC through the miR3655/SURF6/IRF7/IFNβ axis. This provides new potential strategies for treating CRC associated with KRAS mutations or high levels of ETBF.

## Introduction

According to the 2024 cancer statistics, the incidence and mortality rates of colorectal cancer (CRC) are gradually increasing.^1^ Nearly half of the CRC patients exhibit KRAS mutations (MUT).^2,3^ KRAS mutations lead to the continuous activation of the RAS, thereby promoting the proliferation, metastasis, and drug resistance of tumor.^4,5^ KRAS-mutated CRC patients are significantly associated with the worse prognosis.^6^ Although there are currently small-molecule drugs targeting the KRAS G12C mutation,^7^ which accounts for only a small portion of all KRAS mutations.^8^ Moreover, due to issues such as acquired drug resistance, CRC patients have not achieved prolonged survival with KRAS G12C inhibitors.^9–11^ Based on the centrality of KRAS mutations in CRC,^12^ a new therapeutic strategy needs to be developed.

The human gut contains a large number of microbes that influence a variety of physiological processes, including inflammation, immunity, and metabolism.^13,14^ Over the past few decades, it has been established that gut microbes such as Fusobacterium nucleatum (FN), enterotoxigenic bacteroides fragilis (ETBF), and porphyromonas gingivalis (P. gingivalis) promote the proliferation and metastasis of CRC.^14–16^ Given the unique anatomical position of CRC, the close connection between intratumoral microbes and the gut microbiota is inevitable. Understanding the complex interactions between CRC, KRAS mutations, and intratumoral microbes can provide opportunities for the prevention and treatment of tumors.

As we gain a deeper understanding of the oncogenic mechanisms of gut microbiota, it remains unclear how to target and inhibit these oncogenic bacteria to actively improve the prognosis of CRC. Intratumoral microbes are a crucial component of the tumor microenvironment (TME). Modulating intratumoral microbes to enhance the therapeutic response of cancer is a novel concept.^17^ Remodeling the gut microbiota through fecal microbiota transplantation (FMT) can alter the TME, thereby enhancing the efficacy of immune checkpoint inhibitors (ICIs).^18^ Furthermore, studies have indicated that antibiotic treatments can reduce the abundance of oncogenic bacteria, including ETBF and FN, thus decreasing proliferation and tumor growth of CRC in nude mice.^19,20^ However, antibiotics pose their own challenges, such as resistance, disruption of probiotics, and influencing the efficacy of ICI.^21,22^ As a key part of the TME,^23^ developing treatment methods based on specific gut oncogenic bacteria has become a new line of thought.

The association between changes in the gut microbiota and CRC has been extensively confirmed, but the impact of KRAS mutations on the gut microbiota is not clear. We hypothesize that KRAS mutations promote proliferation of CRC by regulating intratumoral microbiota. Initially, this study discovered through metagenomic sequencing that KRAS mutations induce alterations in the function and diversity of the gut microbiota among CRC patients. The most significant changes post-KRAS mutation were in the bacteroidetes. We focused on ETBF, the most classic oncogenic bacteria within the bacteroidetes. The correlation between KRAS mutations and the abundance of ETBF was verified through the fecal genomic DNA of KRAS mutation and KRAS wild type (WT) CRC patients. Through in vitro co-culture assays, bacterial phenotyping assays, and xenografted nude mouse models, we demonstrated that KRAS mutations promote the intratumoral colonization of ETBF in CRC. Mechanistically, KRAS mutations enhance the adhesion and biofilm formation ability of ETBF through the regulation of the miR3655/SURF6/IRF7/IFNβ axis. Therefore, for CRC patients with high levels of ETBF and KRAS mutations, interventions targeting the above axis can be designed.

## Materials and methods

### Clinical samples and collection

According to protocol of research, we prospectively collected fecal, tumor, and adjacent normal tissue from 40 CRC patients after obtaining informed consent. The study was designed to minimize the influences of different regions, dietary habits, and races on metagenomic sequencing and other assays. Forty initially resectable colorectal adenocarcinoma patients admitted to Fujian Provincial Hospital from January 2023 to June 2023, were selected. Fujian Provincial Hospital detected KRAS mutations in tumor tissues by combining quantitative real-time polymerase chain reaction (qPCR) and second-generation sequencing techniques. Patients were divided KRAS MUT (*n* = 20) and KRAS WT (*n* = 20). Inclusion criteria: 1) No special dietary habits; 2) Initially operable primary colorectal adenocarcinoma patients; 3) Age over 18 years old; 4) Histopathological diagnosis as colorectal adenocarcinoma; 5) No distant metastasis. Exclusion criteria: 1) History of other malignant tumors; 2) Secondary CRC; 3) Laxatives taken before fecal collection; 4) Antibiotics used within 6 months before fecal collection; 5) Probiotics consumed within 6 months before fecal collection; 6) Neoadjuvant radiotherapy or chemotherapy performed before surgery; 7) Other gastrointestinal diseases; 8) Any other diseases that might interfere with the results of this research. This study was approved by the ethics committee of Fujian Provincial Hospital. Patient information and KRAS mutation status are shown in Table Supplementary 1 and Table S2.

### Metagenomic sequencing and analysis

Taking the genome of all microorganisms in the feces as the research object, and the DNA of the whole microbial community was performed by high-throughput sequencing. The overall process included genome DNA fragmentation, library construction, quality control, sequencing, and analysis. The integrity, size, and concentration of the library were assessed using 2% agarose gel electrophoresis and the Thermo Qubit 4.0 Fluorometer. High-throughput sequencing was performed on the Illumina™ second-generation sequencing platform. This assay was supported by Sangon Biotech (Shanghai, China).

### Bacterial culture and counting

The standard strains of ETBF (American Type Culture Collection (ATCC) 43859) and Escherichia coli (E. coli) (ATCC25922) were acquired from the ATCC (Manassas, VA, USA). The strains were revived strictly according to the provided instructions. ETBF was anaerobically cultured. Glycerol stocks containing ETBF were stored at −80°C. For revival, a loop was used to streak the bacterial liquid on a blood agar plate. And after 36–48 h, activated single colonies were formed. An activated single colony was then inoculated into BACTEC LYTIC/10 Anaerobic/F Culture Vials and cultured on the shaker (37°C, 200 rpm) for 36 h-48 h. The anaerobic culture bags and gas packs were used for the bacterial phenotype assays, which were obtained from MITSUBISHI GAS CHEMICAL COMPANY, Japan. The number of planktonic bacteria was determined by measuring the optical density (OD) at 600 nm, with an OD (600 nm) = 1 corresponding to an ETBF concentration of approximately 1 × 10^9 cfu/ml.^24,25^ E. coli was cultured in Luria-Bertani medium under aerobic conditions at 37°C.

### Culture and co-culture of CRC cell

The human CRC cell lines (HCT116, LOVO, SW620, SW48, RKO, HT29, DLD-1, and SW480) used were purchased from the ATCC (Manassas, VA, USA). Cell lines cultured for fewer than 16 passages after acquisition were used for the assays. The cells were digested and passaged every 2–3 days depending on the growth condition. Bacteria were inoculated with a multiplicity of infection (MOI) of 1:10 or 1:1. All bacterial phenotype assays required bacteria to be in the logarithmic growth phase.

### Adhesion assay of ETBF

Bacteria and cells were co-cultured (MOI = 1:1). After co-culturing in an anaerobic environment for 3–4 h, the CRC cells were washed three times with 1× sterile PBS to remove non-adherent bacteria. Around 3 min of trypsin digestion was performed for each group, followed by the addition of 600 μL of FBS-containing, antibiotic-free medium to stop the trypsin digestion reaction. Perform gradient dilution using anaerobic culture medium. Take 80 μl of the bacterial suspension and proceed with the spread plate method. Three parallel replicates were set for each group and cultured in an anaerobic environment at 37°C for 48 h. Adhesion ability is represented by the colony-forming units on each plate.^26^ The counts of colonies between 30 and 500 and evenly distributed were considered valid.

### Biofilm formation assay of ETBF

The biofilm formation assay was performed in line with established protocols from previous studies, albeit with a few stepwise modifications.^26–28^ Bacteria and cells were co-cultured anaerobically for 24 h (MOI = 1:1). After co-culturing, the bacterial liquid was diluted and inoculated into a 96-well plate and cultured under anaerobic conditions for 48 h. Each group was repeated five times. The medium was eliminated, and each well was subsequently rinsed five times using 1× sterile PBS. The plate was air-dried for 45–60 min. Each well was stained with 100 μL of 1% crystal violet (CV) solution for 45 min. The staining solution was removed, and the wells were then thoroughly rinsed five times with 1× sterile PBS to ensure the absence of any residual purple coloring. The plate was air-dried for 45–60 min, displaying lightly purple-colored circular biofilms. About 100 μL of 95% ethanol was added to each well to dissolve the biofilms. After 15 min of complete dissolution, OD at 550 nm was detected using the microplate reader.

### Fluorescence in situ hybridization (FISH)

This FISH employed the RNASweAMITM in situ hybridization fluorescence signal detection kit and probe sequences.^26^ Fixative was added to the wells to cover the cells for 15 min. Cells were covered with the permeabilizing agent and incubated at room temperature for 20 min. Proteinase K solution digested cells at 37°C for 15 min. Pre-warmed hybridization solution at 40°C was applied to cover the cell (incubated for 30 min). Pre-warmed hybridization mixes 1 at 40°C was applied to cover the cell (incubated for 12 h). Gradient washed with SSC. Hybridization mixes 2 at 40°C was applied (incubated for 45 min). Gradient washed with SSC. Lastly, pre-warmed fluorescent probe hybridization solution at 37°C was applied (incubated for 45 min). Gradient washed with SSC. After washing once with 1×PBS, DAPI fully covered over the cell (room temperature, protected from light, stained for 8 min). The anti-fluorescence quenching sealing agent was applied. Each sample was scanned at randomly selected locations.

### Interferon-β (IFNβ) bacterial-killing assay

Different concentrations of IFNβ protein and ETBF (1 × 10^8 cfu/ml) were added to 1.5 ml sterile tubes. The tubes were incubated at 37°C under anaerobic conditions (200 rpm). Control samples were incubated in parallel. After corresponding incubation times, the mixture was diluted with anaerobic culture solution by the gradient dilution and using spread plate method on blood plates. The counts of colonies were manually calculated to quantify the survival of ETBF.^29^

### Reverse transcription qPCR (rt-qPCR) and qPCR

RT-qPCR or qPCR was selected according to the design of assay and sample type. For the quantification of ETBF in human feces, this study used qPCR (16S RNA as an internal reference). For the quantification of ETBF in xenograft tumors of nude mice, gDNA was extracted using the QIAamp DNA MiniKit (Qiagen, Germany). The expression of ETBF in the xenograft tumors was detected by qPCR (human prostaglandin transporter (PGT) as the reference gene).^30^ Classic TRIZOL method was used to extract RNA from cells and tumor tissues (GAPDH or U6 as internal reference), and the levels of RNA were detected by RT-qPCR. All assays were carried out with PCR amplification and cycle threshold (CT) value measurement using the Roche Light Cycler 480 system. And the expression level of target genes was calculated using the 2^-ΔΔ CT method (Table S3).

### CCK8 assay

Cells after resuspension were evenly seeded in the 96-well plate. Into each well, 10 μl of CCK-8 solution at a concentration of 5 mg/ml was introduced. The OD at 450 nm was measured using the microplate reader after incubating for 2 h. The assay was repeated 3 times. The above method was used to study the impact of gene expression on proliferation of CRC cell. Additional steps were incorporated to investigate the impact of bacterial presence on cellular behavior. After the cells adhered, the cells were washed three times with 1× sterile PBS. Antibiotic-free medium was added ETBF or E. coli or PBS were co-cultured with cells using an MOI of 10:1.

### Colony formation assay

Cells underwent trypsin digestion and were gently resuspended to achieve a single-cell suspension. A total of 500 cells were plated in each well. The culture process was halted upon the visible emergence of colonies within the dish. Subsequently, the cells were delicately washed with 1× PBS, followed by fixation using 4% paraformaldehyde for 15 min. About 600 μl of 0.1% crystal violet was added per well for 15 min. To study the impact of bacteria on colony formation ability of cells after cell adherence, the antibiotic-free medium was added. ETBF or E. coli or PBS were co-cultured with cells using an MOI of 10:1.

### Enzyme linked immunosorbent assay (ELISA)

The cell culture supernatant (CCS) was harvested from CRC cells in 10 cm culture dish nearing confluence. The concentration of IFNβ secreted (human) in the CCS was detected using the Human Interferon Beta ELISA kit (Abcam, UK), according to the instructions.^31^ Collected CCS was further concentrated using a 10kDa Centrifugal Filter Unit (Millipore, American). The OD at 450 nm was measured.

### Immunofluorescence (IF)

For the various assay groups, cells were fixed using 4% paraformaldehyde and subsequently made permeable with a 0.3% Triton X-100 solution for 10 min. Following this, the cells were shielded with 10% goat serum, then left to incubate overnight at 4°C with anti-IRF7 and anti-SURF6 antibodies. After washes with 1× PBS three times (each lasting 5 min), cells were incubated at room temperature with goat anti-rabbit antibodies or anti-mouse antibodies (diluted 1:200) for 2 h. This was followed by washes with PBS three times (each lasting 5 min) and staining with DAPI (10 mg/ml). Then, images were captured (Zeiss, Germany). Details on the antibodies can be found in Table S4.

### Western blot (WB)

Briefly, the proteins from the cell samples underwent electrophoresis were transferred, blocked, and then incubated at 4°C for 8 h with antibodies. This was succeeded by a 2-h incubation with either goat anti-rabbit IgG or goat anti-mouse IgG. Protein bands were detected using ECL development solution in an imaging system. The grayscale values were measured using Image J software, with the ratio to the internal reference being the final relative expression level.

### Immunohistochemistry (IHC)

IHC is summarized as follows: tissue paraffin embedding, deparaffinization, hydration, blocking endogenous peroxidase activity, heat antigen retrieval, blocking, anti-IFNβ antibody incubation at 4°C, secondary antibody incubation at 37°C, diaminobenzidine staining, hematoxylin counterstaining, and slide sealing for microscopic examination. All results of IHC were independently evaluated by two observers using the staining index (SI).^32^

### Subcutaneous xenograft tumor assay in nude mice

SPF level male BALB/c nude mice (aged 4–5 weeks) were obtained from Shanghai SLAC Laboratory Animal Co., Ltd. All experiments involving nude mice were performed in compliance with the NIH guidelines on the care and use of laboratory animals and received approval from the Animal Use Committee of Fujian Provincial Hospital. Control and experimental groups were established according to the assay arrangements, with five mice per group. For experiments involving intratumoral injections of adeno-associated virus (AAV), mice were randomly divided into groups 7 days after tumor formation. About 5 × 10^6 tumor cells, suspended in 100 μl of PBS, were subcutaneously injected into the right axilla of the respective groups of nude mice. Seven days after tumor formation, 1 × 10^9 CFU/mL of ETBF was injected intratumorally (10 μL per mouse, twice a week for three weeks).^33^ AAV (1 × 10^11 vector genomes (vg)/mouse) were injected intratumorally four times over two weeks after 7 days of tumor formation.^34^ For experiments groups that required antibiotic treatment, mice drank water containing 500 mg/L cefoxitin, while control groups drank PBS. Every four days, the length (L) and width (W) of the tumor were measured using calipers. The volume of the tumor was then calculated with the formula (L×W^2)/2. After four weeks, mice were euthanized. Tumor tissues were collected for qPCR or RT-qPCR analysis.

### Lentivirus and AAV construction

The lentivirus used in this study was formed by a three-plasmid system. The steps summarized include lentivirus vector preparation, lentivirus packaging and purification, lentivirus titer detection, and sterility testing. The lentivirus was used to introduce KRAS G12V, G13D, G12D mutation, and the overexpression (OE) and short hairpin RNA (shRNA) of SURF6. Human CRC cell lines after virus infection (24–48 h) and was followed by 24–48 h of screening by puromycin. The AAV used in this study (including miR3655, SURF6, and IFNβ) was formed by shuttle plasmids carrying the exogenous gene insert, pAAV-RC encoding rep and cap protein encoding genes, and pHelper plasmid. Three plasmids were co-transfected into helper cells AAV-293 to form AAV particles carrying the exogenous insert gene. The AAV was injected into the xenograft tumors of nude mice according to requirements. Both the quality control and preparation of lentivirus and AAV were supported by Genome ditech of Puyun Biotechnology Company (Wuhan, China) (Table S5).

### Dual-luciferase reporter assay

This assay was applied for the confirmation of target genes of miR3655 and the effect on the promoter activities of IRF7 or IFNβ. For the study on the target genes of miR3655, we constructed wild type and mutant 3‘UTR regions (potential-binding sites of miR3655), with the vector being pmirGLO (Promega). For the study on the promoter activities of IRF7 and IFNβ, gene promoter fragments were cloned into PGL4.10 vectors. HEK293T cells, grown in 96-well plates, were co-incubated with the transfection system for 48 h. Each group was set up with three replicates.

### RNA sequencing and analysis

We utilized 3‘end polyA structure of mRNA for RNA sequencing and analysis of this study. The steps briefly summarized include: mRNA isolation, fragmentation, double-stranded cDNA synthesis, modification of cDNA fragments, magnetic bead purification, fragment selection, and library amplification. Following detection and quality assessment, the sequencing library compatible with the Illumina platform was finalized. This assay was supported by Sangon Biotech (Shanghai, China).

### Chromatin immunoprecipitation (ChIP)-qPCR

This ChIP-qPCR used the Pre-ChIP Assay Preparation Guidelines for ChIP Kit (Magnetic, qPCR) (Abcam, UK) for DNA fragment purification and qPCR analysis. The ChIP-qPCR of this study was used to explore whether SURF6 can directly bind to IRF7.

### mRNA stability

To assess RNA stability, Actinomycin D (Act-D, HY-17559, MCE, USA) was applied. After incubation for varying durations, RNA was extracted for RT-qPCR. The half-life of SURF6 mRNA was determined and normalized to the internal reference GAPDH.

### Bioinformatics analysis

For bioinformatics analysis, public genomic data from the Gene Expression Omnibus (GEO) or the Cancer Genome Atlas (TCGA) were applied in this study. All sample expression levels of mRNA (expressed as counts per million [CPM]) were analyzed using the R version 4.2.2.

### Statistical methods

All data were statistically analyzed using SPSS 25.0 software, GraphPad Prism version 9.5.1, and R programming language. The mean comparisons between two groups for continuous variable were performed using Student’s t-test or Wilcoxon rank-sum test. Multi-group mean analysis was conducted using One-Way Analysis of Variance (ANOVA). *p* < 0.05 was considered statistically significant.

## Results

### KRAS mutations alter the composition and diversity of the gut microbiota in CRC

**Figure 1.**
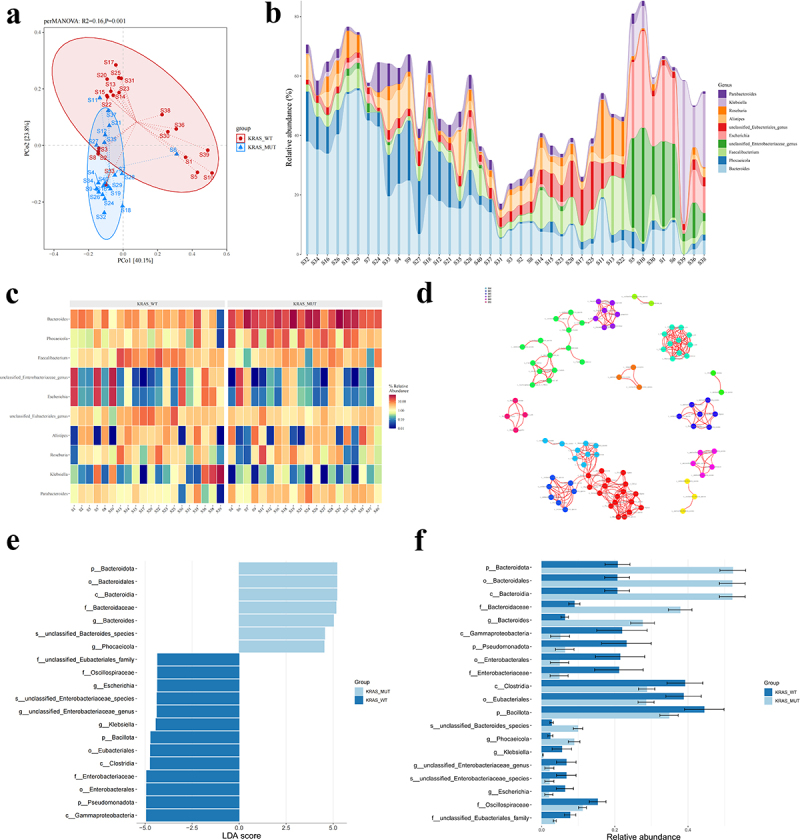
KRAS mutations alter the composition and diversity of the gut microbiota in CRC.

To investigate whether KRAS mutations affect the composition and diversity of the gut microbiota in CRC patients, we performed metagenomic sequencing on fecal samples prospectively collected from CRC patients with KRAS mutations (*n* = 20) and KRAS WT (*n* = 20) (Figure S1A and Table S1-S2). Initially, principal coordinates analysis (PCoA) and Bray-Curtis distance box plots clearly distinguished the gut microbiota of all samples between KRAS mutations and KRAS WT (Figure 1(a) and Figure S1b). The fecal microbiota of KRAS mutations and KRAS WT CRC patients were separated into two clusters (*p* = 0.001, perMANOVA). This indicates that in CRC, compared to KRAS WT gut microbiota, KRAS mutations have different diversity and microbial distance metrics. After species annotation, we obtained the abundance of gut microbiota. Figure 1(b) shows an overview at the level of genus for 40 CRC patients, revealing differences in the gut microbiota of each patient. We performed quartile calculations for the dominant species with high abundance (Figure S1c) and found that the abundance of Bacteroides and Phocaeicola positively correlated with KRAS mutations. Similarly, the abundance heatmap also suggested that the expression levels of Bacteroides and Phocaeicola are elevated in the feces of CRC patients with KRAS mutations (Figure 1(c)). This initial observation suggests a difference in the gut microbiota between CRC patients who have KRAS mutations and those with KRAS WT. The interactions among gut microbiota are closely related to the development of tumors, and we further explored the correlation analysis network in two groups of dominant species (Figure 1d). We discovered a clustering effect among dominant bacteria between CRC patients with KRAS mutations and KRAS WT, which Bacteroides interacting with various bacteria.

(a) Principal coordinate analysis (PCoA) of the gut microbiota from fecal samples of CRC patients with KRAS WT versus KRAS MUT (20 vs. 20; *p* = 0.001). (b) Relative abundance of genus impact bar graph. Provides a clear observation of the distribution trends of dominant genus across all samples. (c) Abundance heatmap. Each row represents a genus, each column represents a sample, and the color reflects the relative abundance of the genus in the sample. A redder color indicates higher abundance, while a bluer color indicates lower abundance. (d) Correlation network analysis. By analyzing the correlation between species and abundance information across different samples, we identify the similarity, coexistence probability, and clustering effect between species and functions, thus uncovering the interrelationships between species and functions. (e) Linear discriminant analysis (LDA) coupled with effect size analysis (LEfSe). LDA is used to estimate the impact of these differential microbiota on the differences between groups. Different colors represent different groups, the horizontal axis shows the LDA score obtained after LDA analysis, and the vertical axis shows the significant microbial taxa. (f) Abundance difference bar graph of inter-group. The vertical axis represents species (including Phylum, Class, Order, Family, Genus, Species) marked as Lefse-marker, and the horizontal axis represents relative abundance, with differently colored bars representing the relative abundance in different groups. (*p* > .05, ns. nonsignificant; *p* < .05 *; *p* < .01 **; *p* < .001 ***; *p* < .0001 ****; Analysis performed using Student’s t-test, Wilcoxon rank-sum test, or Welch's ANOVA test for single or grouped analyses, respectively)

To quantitatively screen for the gut microbes with the most significant abundance changes in CRC patients with KRAS mutation, we conducted the linear discriminant analysis coupled with effect size analysis (LEfSe). We identified the microbes that could explain inter-group differences and the degree of their influence on these differences (Figure 1e). We further conducted hierarchical annotation and visualization analysis with GraPhlAn based on the genus abundance of samples to select dominant genus (Figure S1d). Combining species and functional hierarchy information for visualization and annotation. Integrating LEfSe and GraPhlAn visualization analysis (Figure 1(e) and Figure S1D), we observed that Bacteroidota and Gammaproteobacteria exhibited the most pronounced differences in their impact on the gut microbiota of the two groups. The LEfSe marker inter-group abundance difference bar chart further validated that the abundance level of Bacteroides was found to have a positive correlation with KRAS mutations (Figure 1(f)), similar to previous detection results of intratumoral microbial.^35^ The abundance of Gammaproteobacteria, Pseudomonadota, and Enterobacterales decreased in the KRAS mutation group (Figure 1(f)). In conclusion, the diversity and composition of the gut microbiota in CRC patients with KRAS mutations have changed significantly, which Bacteroides positively correlated with KRAS mutations.

### KRAS mutations facilitate the intratumoral colonization of ETBF in CRC

**Figure 2.**
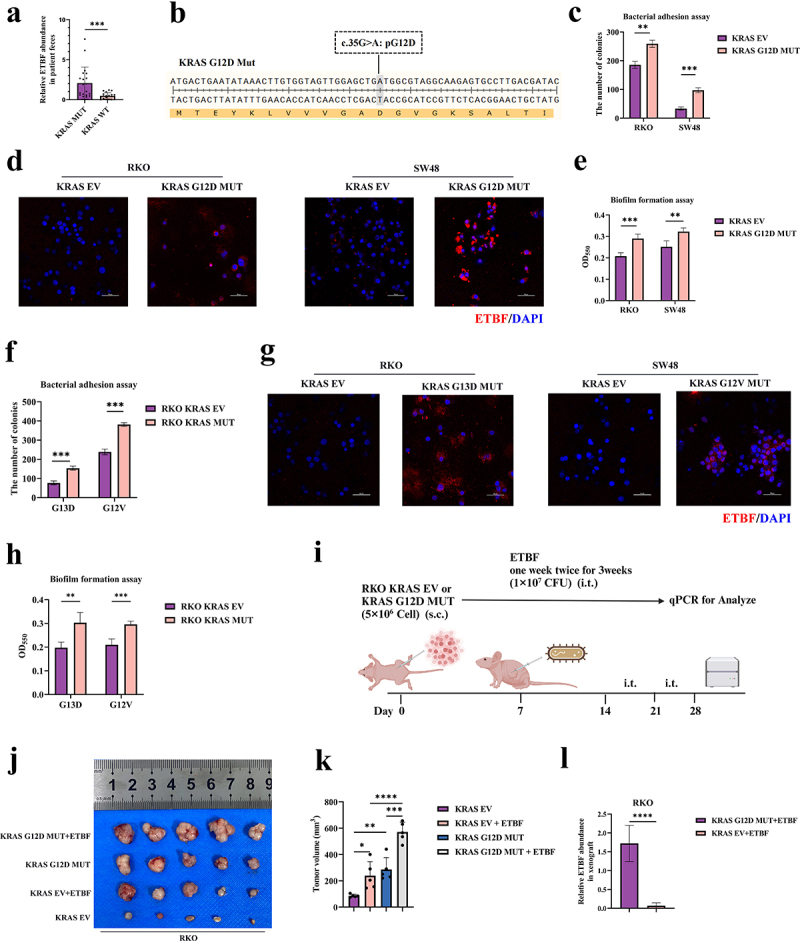
KRAS mutations promote the intratumoral colonization of ETBF in CRC.

ETBF, a classic carcinogenic bacterium within the Bacteroides genus, has been extensively studied for its role in promoting proliferation, stemness, and metastasis of CRC.^16,26,33^ Integrating results of metagenomics, we hypothesized that KRAS mutations might facilitate the intratumoral colonization of ETBF in CRC, further enhancing carcinogenic effects. Based on the results of metagenomics, we found that in CRC, KARS mutation was associated with elevated abundance of ETBF (Figure S1e). To verify this finding, we extracted the gut microbial genomes from the feces of CRC patients using fecal genome DNA kits. Through qPCR, we found that the abundance of ETBF in the feces of 40 CRC patients positively correlated with KRAS mutations (Figure 2a), supporting the findings from metagenomics. KRAS G12D is the most common mutation site in CRC.^36,37^ To ascertain whether KRAS mutations could promote colonization of ETBF in CRC at the cellular level, we constructed CRC cell models with KRAS EV and corresponding KRAS G12D MUT in KRAS WT CRC cell lines (SW48 and RKO) (Figure 2(b)). Compared to KRAS EV, KRAS G12D MUT cells showed a faster rate of proliferation and greater colony formation ability (Figure S2a-S2b). To further confirm the success of the KRAS G12D MUT cell models, we performed sanger sequencing and found that the mutation in codon 12 replaced glycine with aspartic acid (Figure S2c). Next, we co-cultured ETBF and CRC cell lines. We found that ETBF could promote proliferation and stemness of CRC cells (Figure S2d-S2e), verifying again that ETBF is a carcinogenic bacterium.^33,38^

(a) A combined fecal genomic DNA kit and qPCR method to quantify the abundance of ETBF in the feces of CRC patients with KRAS mutations and KRAS WT, qPCR: quantitative real-time polymerase chain reaction. (b) Schematic model of KRAS G12D MUT cells. (c) The impact of KRAS G12D MUT on the adhesive ability of ETBF. (d) Fluorescence in situ hybridization (FISH) analysis of ETBF co-cultured with KRAS G12D MUT cells, repeated three times for each group. (e) The influence of KRAS G12D MUT on biofilm formation capabilities of ETBF. (f) The effect of KRAS G12V MUT and KRAS G13D MUT on the adhesive ability of ETBF. (g) FISH analysis of ETBF co-cultured with KRAS G12V MUT and KRAS G13D MUT cells, repeated three times for each group. (h) The impact of KRAS G12V MUT and KRAS G13D MUT on biofilm formation abilities of ETBF. (i) Schematic of animal experiment (xenograft tumors of nude mice). s.c.: subcutaneous injection; i.t.: intratumoral injection. (j) Images of xenograft tumors of nude mice from different experimental groups (5 mice per group). (k) Volumes of xenograft tumors on day 28 in nude mice from different experimental groups (5 mice per group). (l) qPCR analysis of the relative abundance of ETBF in xenograft tumors from different experimental groups (5 mice per group). (*p* > .05, ns; *p* < .05 *; *p* < .01 **; *p* < .001 ***; *p* < .0001 ****; Analysis performed using Student’s t-test, Wilcoxon rank-sum test, or Welch’s ANOVA test for single or grouped analyses, respectively)

To confirm our hypothesis, we first conducted bacterial adhesion assays. After co-culturing bacteria and cells, we confirmed that compared to KRAS EV, KRAS G12D MUT could promote the adhesion of ETBF in CRC (Figure 2(c)). The FISH assay also validated this finding (Figure 2(d)). KRAS G12D mutations significantly enhanced the intratumoral colonization ability of ETBF in CRC. The biofilm formation ability of ETBF, which is a widely noted characteristic of carcinogenic bacteria, consisting of structures formed by bacteria through secretion, provides protection for bacteria, enhances nutrient acquisition, and promotes inter-bacterial communication.^39,40^ We confirmed that KRAS G12D MUT also enhanced the biofilm formation ability of ETBF in CRC (Figure 2(e)). Adhesion and biofilm formation abilities are crucial for microbes to colonize tumor cells. In summary, in vitro assay level, KRAS G12D mutations can promote the intratumoral colonization of ETBF in CRC.

To determine whether the impact of KRAS mutations on the intratumoral colonization ability of ETBF in CRC was limited to the KRAS G12D site, we also constructed additional common KRAS mutation sites in CRC (Figure S2f).^36^ In comparison to KRAS EV, KRAS G13D MUT and KRAS G12V MUT significantly enhanced the adhesion ability of ETBF in CRC (Figure 2(f)). FISH further validated that KRAS G13D MUT and KRAS G12V MUT enhanced the intratumoral colonization of ETBF in CRC (Figure 2(g)). Consistent with our expectations, KRAS G13D MUT and KRAS G12V MUT also increased the biofilm formation ability of ETBF in CRC (Figure 2(h)).

To validate how KRAS mutations affect the interaction between microbes and tumor cells in complex organisms, we intratumorally injected ETBF into xenografted nude mouse tumors (Figure 2i). As shown in Figure 2(j,k) and Figure S2g-S2h, KRAS G12D MUT had notably faster growth rate, higher tumor weight, and volume compared to KRAS EV. Moreover, intratumoral injection of ETBF significantly enhanced proliferation of CRC. We extracted gDNA from the xenografts and confirmed through qPCR that the abundance of ETBF was higher within KRAS G12D MUT tumors (Figure 2(I)), validating the findings from in vitro assay. In conclusion, this section verified that ETBF promotes proliferation and stemness of CRC. We discovered that KRAS mutations, both in vitro and in vivo, promote intratumoral colonization of ETBF in CRC for the first time. Moreover, the ability of KRAS to promote intratumoral colonization of ETBF is not limited to the G12D site, while other common KRAS mutation sites also play a role.

### KRAS mutations suppress the expression of miR3655 in CRC

**Figure 3.**
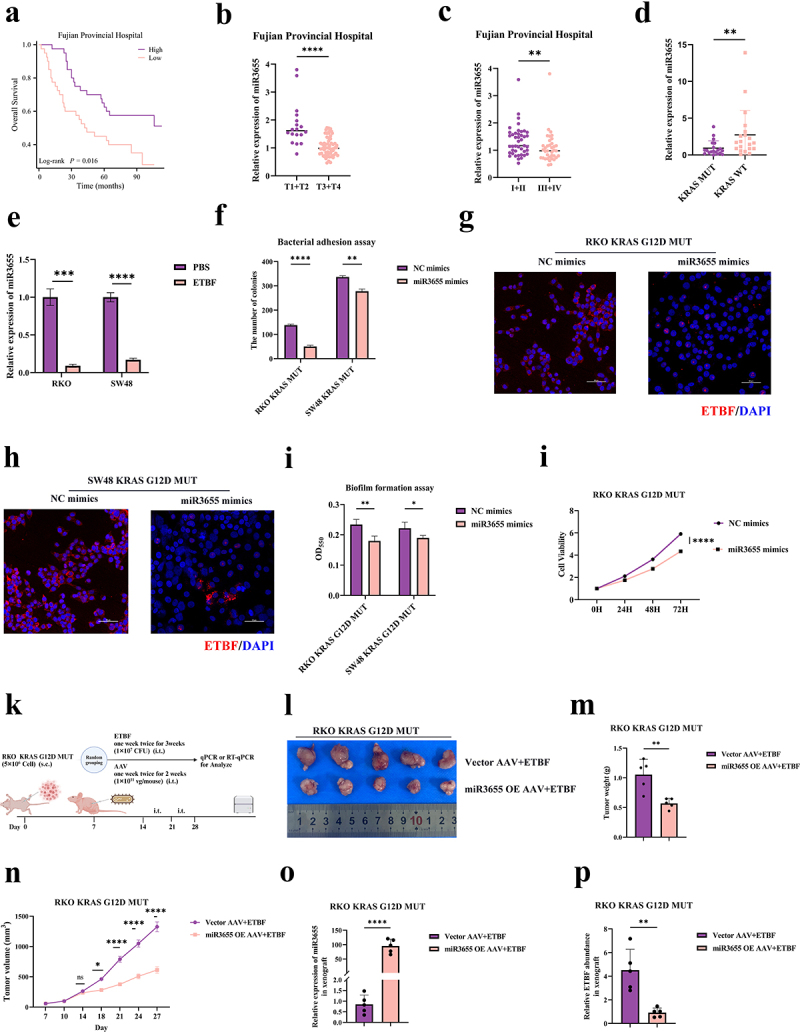
KRAS mutations promote intratumoral colonization of ETBF in CRC by inhibiting miR3655.

After confirming that KRAS mutations promote the intratumoral colonization of ETBF in CRC, we became curious about the specific mechanisms involved. Since microRNA (miRNA) can mediate the regulation of interspecies genes and thus affect the control of the host over the gut microbiota,^41^ we turned our attention to miRNA. We explored the GEO database for differentially expressed miRNA associated with KRAS mutations (GSE72577). KRAS mutation and CRC are closely related to chronic intestinal inflammation.^42,43^ In addition, ETBF stimulates chronic intestinal inflammation, which contributes to the development and progression of CRC.^44^ Hence, we also searched for miRNA related to colitis (GSE121608). We discovered that miR3655 is the only miRNA present in both datasets (Figure S3a). Through TCGA database, we confirmed that the expression level of miR3655 in CRC tumor tissues is lower compared to normal tissues (Figure S3b). Furthermore, the receiver operating characteristic (ROC) curve identified miR3655 as one of the biomarkers for predicting CRC (Figure S3c). Currently, miR3655 in CRC remains unstudied. Utilizing survival data from 80 patients at Fujian Provincial Hospital and applying the Kaplan–Meier (KM) survival analysis curve, we found that the high expression of miR3655 is significantly correlated with the better overall survival (OS) in CRC patients (Figure 3(a)). We further explored the relationship between miR3655 and clinical staging, finding that the low expression of miR3655 is linked to more advanced T stage, N stage, and TNM stage (Figure 3(b,c) and S3d). This suggests that the expression of miR3655 is inversely related to proliferation, metastasis, and invasion of CRC. miR3655 may play a tumor-suppressive role in CRC; however, the specific mechanisms by which miR3655 exerts its anti-cancer effects are currently unclear.

(a) Kaplan–Meier (KM) survival analysis on overall survival (OS) of CRC patients with high versus low expression of miR3655 (*n* = 80 CRC patients). (b) Expression of miR3655 in CRC patients across different T stages (*n* = 80 CRC patients). (c) Expression of miR3655 in CRC patients across different TNM stages (*n* = 80 CRC patients). (d) Correlation between expression levels of miR3655 and KRAS mutation in prospectively collected CRC tumor tissues (*n* = 40 CRC patients). (e) RT-qPCR detection of expression levels of miR3655 after co-culturing cells with ETBF or PBS (control); RT-qPCR: Reverse Transcription-qPCR. (f) The effect of miR3655 mimics the adhesive ability of ETBF in CRC cells. (g and h) FISH analysis of ETBF co-cultured with KRAS G12D MUT CRC cells treated with miR3655 mimics, repeated three times for each group. (i) The impact of miR3655 mimics the biofilm formation capability of ETBF in CRC cells. (j) CCK-8 assay to assess the effect of miR3655 mimics on the proliferation of CRC cells. (k) Schematic of animal treatment (xenograft tumors of nude mice). s.c.: subcutaneous injection; i.t.: intratumoral injection. (l) Images of xenograft tumors of nude mice from different experimental groups (5 mice per group). (m) Weights of xenograft tumors were measured at the end of the experiment in different experimental groups (5 mice per group). (n) Growth curves of xenograft tumors of nude mice across different experimental groups (5 mice per group). (o) Detection of expression levels of miR3655 in xenograft tumors after intratumoral injection of AAV by RT-qPCR. (p) qPCR analysis of the relative abundance of ETBF in xenograft tumors from different experimental groups (5 mice per group). (*p* > .05, ns; *p* < .05 *; *p* < .01 **; *p* < .001 ***; *p* < .0001 ****; Analysis performed using Student’s t-test, Wilcoxon rank-sum test, or Welch’s ANOVA test for single or grouped analyses, respectively)

After clarifying the relationship between miR3655 and CRC, we delved deeper into examining the association between the expression levels of miR3655, KRAS mutations, and ETBF. As shown in Figure 3(d), compared to 20 KRAS WT CRC tumor tissues, the expression level of miR3655 is reduced in 20 KRAS mutant. More interestingly, a decrease in the expression level of miR3655 was also found in post-ETBF infection CRC cells (Figure 3(e)). Not only in tissues, we also verified the correlation between miR3655 and KRAS mutations in 8 commercial CRC cell lines (6 KRAS MUT/2 WT) (Figure S3e). Focusing subsequent assays mainly on the KRAS G12D MUT cell model, which is the most common mutation site in CRC. We also noticed a reduction in the expression level of miR3655 in the KRAS G12D MUT cell model (Figure S3f). In summary, as a new molecule that can predict diagnosis and prognosis of CRC, KRAS mutations, and ETBF infection both suppress the expression of miR3655 in CRC.

### miR3655 is involved in KRAS mutation-promoted intratumoral colonization of ETBF in CRC

To observe whether miR3655 can affect the intratumoral colonization of ETBF in CRC, we transfected miR3655 inhibitors into KRAS WT cell and miR3655 mimics into KRAS mutant cells (Figure S3g-S3h). As shown in Figure 3f and Figure S3i, miR3655 mimics in KRAS MUT cells significantly inhibited the adhesion ability of ETBF in CRC. In contrast, suppressing the levels of miR3655 in KRAS EV cells significantly promoted the adhesion ability of ETBF. FISH showed that after an increased expression of miR3655, the colonization of ETBF inside and around KRAS-mutant CRC cells was significantly reduced (Figure 3(g,h)). Suppressing the expression of miR3655 in KRAS EV cells significantly enhanced the colonization ability of ETBF (Figure S3j). Additionally, altering the expression of miR3655 in CRC cells also significantly affected the biofilm formation ability of ETBF (Figure 3i and S3k). It is evident that the levels of miR3655 significantly affect the colonization ability of ETBF in CRC. Apart from inhibiting the colonization ability of ETBF in CRC, we were intrigued to assess whether miR3655 could also influence the proliferative capability of CRC cells. CCK8 assay confirmed that miR3655 also influences the proliferative capability of CRC (Figure 3(j) and S3l).

Having established that miR3655 inhibited the intratumoral colonization ability of ETBF in vitro, we overexpressed miR3655 in subcutaneous xenografted tumors in nude mice to observe whether miR3655 could affect the intratumoral colonization ability of ETBF (Figure 3(k)). As shown in Figure 3l-o and S3M, after applying miR3655 OE AAV in KRAS G12D MUT xenografted tumors, the growth rate, weight, and volume of the tumor were significantly reduced. qPCR of the xenografted tumors confirmed that miR3655 inhibited the abundance of ETBF in CRC (Figure 3(p)). This validates our findings from in vitro assays. In summary, KRAS mutations promote the proliferation of CRC and the intratumoral colonization of ETBF in CRC by suppressing the expression of miR3655.

### miR3655 targets the KRAS mutation-associated molecule SURF6 in CRC

**Figure 4.**
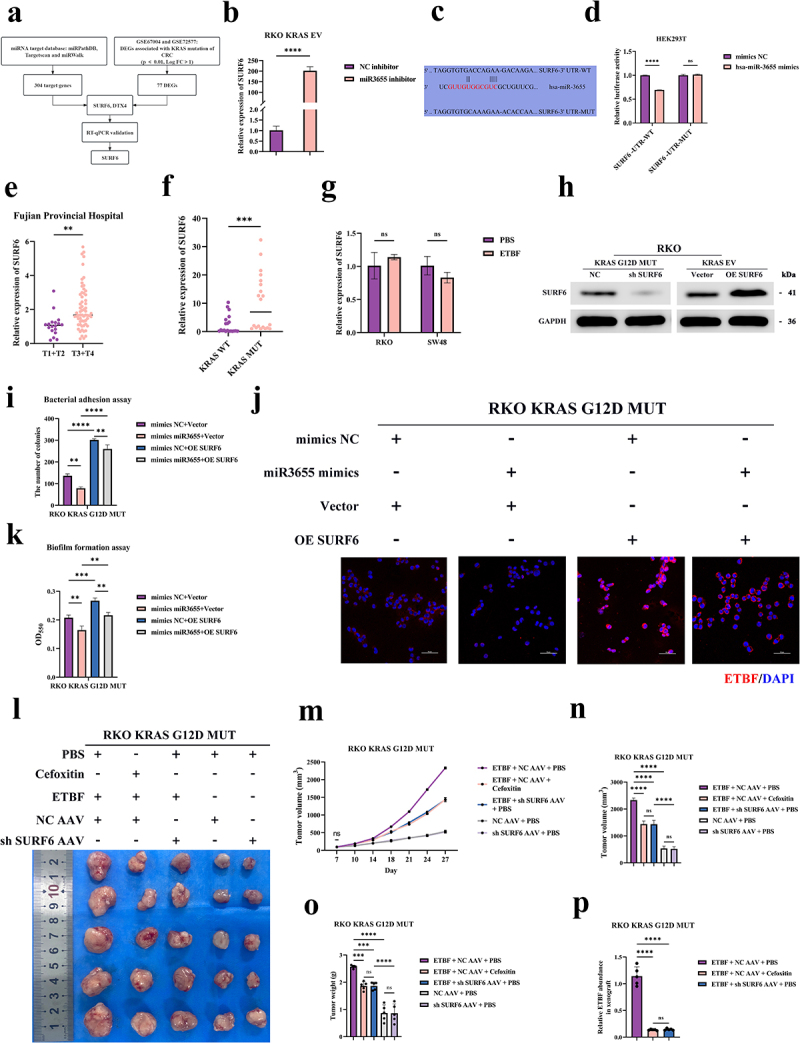
miR3655 inhibits intratumoral colonization of ETBF in CRC by targeting the KRAS mutation-associated molecule SURF6.

To clarify the downstream mechanism by which miR3655 inhibits the colonization of ETBF, we used GEO datasets and bioinformatic strategies to search for candidate targets. First, we analyzed three widely used miRNA target gene databases: miRPathDB, Targetscan, and miRWalk. We then analyzed differentially expressed mRNA related to KRAS mutations (GSE67004 and GSE72577). We identified two predicted genes that met the above criteria, including SURF6 and DTX4 (Figure 4(a)). Through RT-qPCR, we found that transfection with miR3655 inhibitor significantly upregulated the mRNA levels of SURF6, while the mRNA expression levels of DTX4 did not change significantly (Figure 4(b) and Figure S4a). Furthermore, we discovered that the expression level of SURF6 was markedly reduced after transfection with miR3655 mimics (Figure S4b and S4c). From this, we infer that SURF6 is likely a target of miR3655. We cloned the sequence near the 3‘UTR of SURF6 for the dual-luciferase reporter assay and constructed the corresponding mutant vector (Figure 4(c)). When miR3655 mimics were transfected into HEK293T cells, the luciferase activity of the WT SURF6 3‘UTR was reduced (Figure 4(d)). Conversely, when the mutant SURF6 3‘UTR was co-transfected with miR3655 mimics, the reduction in luciferase activity was abolished. In summary, these data suggest that SURF6 may be a direct target of miR3655.

(a) Utilizing the GEO database to identify KRAS mutation-related mRNAs in CRC, intersecting with target genes from the miRNA databases. SURF6 was confirmed by RT-qPCR. (b) RT-qPCR to measure the expression level of SURF6 in CRC after treated with miR3655 inhibitors. (c) Prediction of the binding sites between miR3655 and SURF6. (d) Dual-luciferase reporter assay for the co-transfection of SURF6-UTR-WT/MUT and miR3655 mimics in HEK293T cells (*n* = 3). (e) Expression of SURF6 among CRC patients across different T stages (*n* = 80 CRC patients). (f) Correlation between expression levels of SURF6 and KRAS mutation status in prospectively collected CRC tumor tissues (*n* = 40 CRC patients). (g) Detection of expression of SURF6 after co-culturing cells with ETBF or PBS (control) by RT-qPCR. (h) WB for the protein levels of SURF6 in CRC cells after silencing and overexpressing (OE) SURF6. (i) The effect of OE SURF6 on the adhesive ability of ETBF in CRC cells after treated with miR3655 mimics. (j) After treating cells with miR3655 mimics and subsequently OE SURF6, these cells were co-cultured with ETBF. FISH analysis was performed, with each group repeated three times. (k) The impact of OE SURF6 on the biofilm formation capability of ETBF in CRC cells after miR3655 mimics treatment. (l) Images of xenograft tumors of nude mice from different experimental groups (5 mice per group). (m) Growth curves of xenograft tumors of nude mice across different experimental groups (5 mice per group). (n) The volume of xenograft tumors on day 28 in different experimental groups of nude mice (5 mice per group). (o) Weights of xenograft tumors were measured at the end of the experiment in different experimental groups (5 mice per group). (p) qPCR analysis of the relative abundance of ETBF in xenograft tumors from different experimental groups (5 mice per group). (*p* > .05, ns; *p* < .05 *; *p* < .01 **; *p* < .001 ***; *p* < .0001 ****; Analysis performed using Student’s t-test, Wilcoxon rank-sum test, or Welch’s ANOVA test for single or grouped analyses, respectively)

Since SURF6 has not been studied in cancers, we investigated the correlation between SURF6 and CRC. Using the TCGA database, we found that compared to normal tissues, the expression level of SURF6 is significantly elevated in CRC tumor tissues (Figure S4d). A ROC Curve analysis revealed that SURF6 is one of the biomarkers for predicting CRC (Figure S4e). The KM survival analysis of 80 patients from Fujian Provincial Hospital revealed that the high expression of SURF6 is an adverse factor for OS in CRC (Figure S4f). In addition to correlation with prognosis, the high expression of SURF6 is also related to the advanced T stage, N stage, and TNM stage in CRC (Figure 4(e) and S4g). Thus, SURF6, as a new marker, may predict the prognosis of CRC.

After clarifying the relationship between SURF6 and CRC, we further investigated the role of SURF6 in KRAS mutations and ETBF. Compared to 20 KRAS WT, the expression level of SURF6 is higher in the 20 KRAS mutant CRC tumor (Figure 4(f)). Unlike miR3655, the expression of SURF6 did not change in CRC cells after ETBF infection (Figure 4(g)). This suggests that the effect of ETBF on miR3655 may not be achieved by targeting SURF6. Apart from correlation with KRAS mutations in CRC tissues, we observed an increase in expression of SURF6 in our KRAS G12D MUT cell model (Figure S4h). In summary, as a new biomarker that can predict the prognosis of CRC, the expression level of SURF6 is increased after KRAS mutations. After ETBF infection, there is no significant change in the expression level of SURF6.

### miR3655 inhibits the intratumoral colonization of ETBF in CRC by targeting SURF6

To investigate whether miR3655 affects the colonization capability of ETBF in CRC by targeting SURF6, we first constructed cell lines with stable OE and sh SURF6. The success of the stable transfection was confirmed via WB analysis (Figure 4(h) and S4i). The inhibition expression levels of SURF6 by miR3655 was restored through OE SURF6 (Figure S4j). The “rescue” assay indicated that OE SURF6 could restore the adhesion ability of ETBF weakened by high expression of miR3655 (Figure 4(i) and S4k). The negative regulatory relationship between SURF6 and miR3655 was further supported by FISH (Figure 4(j) and S4l). The miR3655 mimics led to a decrease of ETBF inside and around the cells and significantly increased after OE SURF6. The inhibitory effect of miR3655 mimics on colonization of ETBF could be rescued by OE SURF6 (Figure 4(j) and S4l). Through Figure 4(k) and S4m, we also noted that OE SURF6 promotes the biofilm-forming capability of ETBF. Furthermore, the negative relationship between miR3655 and SURF6 was reconfirmed by TCGA (Figure S4n). These rescue assays provide additional evidence supporting the theory that SURF6 is a direct and functional target of miR3655. Additionally, we investigated whether SURF6 could directly affect proliferation of CRC and exert a carcinogenic effect, similar to miR3655. Through CCK8 proliferation assays, we found that solely stable OE SURF6 did not affect proliferation of CRC (Figure S4o). This indicates that the direct inhibition of proliferation of CRC by miR3655 is achieved by targeting other mRNA. From these in vitro assays, it is observed that SURF6 promotes the proliferation of CRC by enhancing the intratumoral colonization capability of ETBF.

To verify whether SURF6 can promote the colonization of ETBF in CRC in vivo, we randomly divided the nude mice one-week after tumor formation. According to the assay arrangement, treatment was conducted with oral administration of cefoxitin and intratumoral injection of AAV. The in vivo assays affecting colonization of ETBF revealed that merely inhibiting SURF6 could not suppress the growth rate, tumor volume, and tumor weight of CRC (Figure 4(l-o) and S4p). Compared with solely intratumoral injection of ETBF, inhibiting SURF6 in tumors containing ETBF could suppress proliferation of CRC, which validated the findings from in vitro assays. Additionally, inhibiting SURF6 appeared to have a consistent therapeutic effect with oral administration of cefoxitin. The proliferative effect of ETBF on CRC was once again validated. Astonishingly, further analysis of the subcutaneously xenograft tumor samples from the nude mice revealed that silencing SURF6 significantly reduced the intratumoral abundance of ETBF (Figure 4(p)). In summary, KRAS mutations weaken the targeted inhibitory effect on SURF6 by suppressing miR3655. All the above assays confirmed that SUFR6 promotes the carcinogenic effects by facilitating the intratumoral colonization of ETBF in CRC.

### SURF6 suppresses the Toll-like receptor signaling pathway and the expression of IRF7/IFNβ in CRC

**Figure 5.**
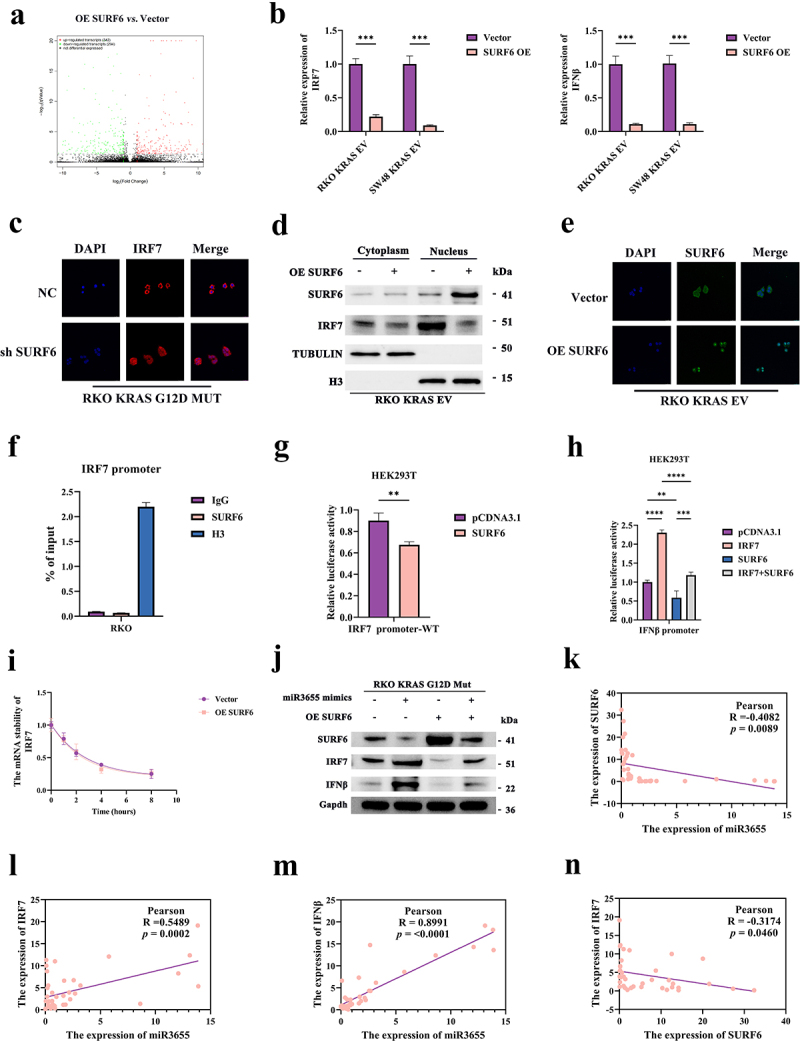
SURF6 suppresses the Toll-like receptor signaling pathway and the expression of IRF7/IFNβ in CRC.

After determining the role of miR3655/SURF6 in ETBF, we became curious about how miR3655/SURF6 affects the downstream mechanism of intratumoral colonization of ETBF in CRC. Transcriptome RNA-Seq was performed on SW48 cells with vector control and OE SURF6 (Figure 5(a)). Among all genes, we identified 636 differentially expressed genes (DEGs; |log2 fold change (FC)|>1, q Value <0.05), including 294 downregulated genes and 342 upregulated genes (Figure 5a). Analysis of downregulated DEGs within the KEGG pathways revealed that OE SURF6 inhibited immune-related signaling pathways such as the “Toll-like receptor (TLR) signaling pathway” and the “NOD-like receptor signaling pathway” (Figure S5a). KEGG pathway analysis of upregulated DEGs showed that OE SURF6 activated pathways like “Biosynthesis of unsaturated fatty acids” and others (Figure S5b). Among them, the TLR signaling pathway was the most significantly influenced pathway by SURF6 regulation (Figure S5a), which is consistent with our previous assays and expectations. As is well known, the TLR signaling pathway acts as the “gatekeeper” of the human immune system, protecting the host from bacterial and other microbial invasions.^45,46^ In summary, these analyses suggest that miR3655/SURF6 may promote intratumoral colonization of ETBF in CRC by regulating the TLR signaling pathway.

(a) Differential expression of volcano plot. Each point represents a gene. Red indicates upregulated genes, green indicates downregulated genes, and black indicates non-differential genes. (b) Detection of the expression of IRF7 and IFNβ in CRC cells after OE SURF6 by RT-qPCR. (c) Detection of expression (red) of IRF7 through immunofluorescence (IF) after short hairpin (sh) SURF6 in CRC cells; DAPI (blue) shows the nucleus. (d) Detection of the expression of SURF6 and IRF7 in the subcellular compartments of CRC cells after OE SURF6 through WB. (e) Detection of the expression of SURF6 (green) through IF after OE SURF6 in CRC cells; DAPI (blue) shows the nucleus. (f) Detection of the binding between SURF6 and the IRF7 promoter by ChIP-PCR. (g) Dual-luciferase reporter assay in HEK293T cells co-transfected with the IRF7 promoter and either a negative control plasmid or SURF6 plasmid (*n* = 3). (h) Dual-luciferase reporter assay in HEK293T cells co-transfected with the IFNβ promoter and either a negative control plasmid or SURF6 plasmid or IRF7 plasmid (*n* = 3). (i) RNA stability assay. Cells were treated with the RNA transcription inhibitor Act-D at different times. (j) Detection of the expression levels of IRF7 and IFNβ through WB after OE SURF6 in CRC cells treated with miR3655 mimics. (k) Pearson analysis of the correlation between miR3655 and SURF6 of CRC patients in Fujian Provincial Hospital (*n* = 40). (l) Pearson analysis of the correlation between miR3655 and IRF7 of CRC patients in Fujian Provincial Hospital (*n* = 40). (m) Pearson analysis of the correlation between miR3655 and IFNβ of CRC patients in Fujian Provincial Hospital (*n* = 40). (n) Pearson analysis of the correlation between SURF6 and IRF7 of CRC patients in Fujian Provincial Hospital (*n* = 40). (*p* > .05, ns; *p* < .05 *; *p* < .01 **; *p* < .001 ***; *p* < .0001 ****; Analysis performed using Student’s t-test, Wilcoxon rank-sum test, or Welch's ANOVA test for single or grouped analyses, respectively)

Transcriptome RNA-Seq identified DEGs in the TLR signaling pathway, including interferon regulatory factor 7 (IRF7), IL6, and CXCL8. Among them, IRF7, as a significant gene in the TLR signaling pathway, garnered our attention. IRF7 has a synergistic effect with downstream IFNβ. And transcription activation of IFNβ requires an enhancer containing IRF7,^47–49^ Furthermore, all elements of immunological function of IFNβ are controlled by IRF7.^50^ IRF7/IFNβ plays a crucial role in enhancing chemotherapy efficacy, preventing tumor recurrence, and boosting immune effects in CRC.^51,52^ In addition, IFNβ has been proven not only to activate immune cells but also to exhibit direct antimicrobial activity against staphylococcus aureus.^53^ However, whether IRF7/IFNβ can inhibit intratumoral colonization of ETBF in CRC remains unclear. For this, we mapped all DEGs to the corresponding protein interaction network, selecting key genes based on topological properties within the network. Ultimately, we found that IRF7 plays a central role among all DEGs (Figure S5c).

Based on a comprehensive sequencing and literature review, we hypothesize that SURF6 promotes intratumoral colonization of ETBF in CRC by inhibiting IRF7/IFNβ. Initially, RT-qPCR discovered that OE SURF6 led to the suppression of IRF7 and IFNβ expression (Figure 5(b)), whereas silencing SURF6 resulted in increased expression levels of IRF7 and IFNβ (Figure S5d). This preliminary finding of a negative correlation between SURF6 and expression of IRF7/IFNβ aligns with the results from transcriptomic sequencing. Currently, how SURF6 regulates IRF7 remains unclear. As a transcription factor, IRF7 needs to enter the nucleus to further activate functions. Hence, we are the first to perform IF of nuclear translocation assays. In the negative control (NC) group, IRF7 was located both in the cytoplasm and nucleus (Figure 5(c)). Silencing SURF6 led to a significant increase in expression of IRF7 and nuclear aggregation, indicating that inhibiting SURF6 promotes the nuclear translocation activation of IRF7. This phenomenon was validated in SW48 KRAS G12D MUT cells (Figure S5e). Further, subcellular partitioning and WB confirmed that OE SURF6 significantly inhibits the nuclear aggregation of IRF7 (Figure 5(d) and S5f). These results lead us to believe that SURF6 can regulate the nuclear translocation of IRF7 in CRC cells. Additionally, we found that OE SURF6 significantly promotes a high degree of SURF6 aggregation in the cell nucleus (Figure 5(d) and S5f). Further, IF verified these findings (Figure 5(e) and S5g). Hence, we speculate whether SURF6 acts as an unverified new transcription factor directly binding to IRF7. Through ChIP-qPCR assay, we discovered SURF6 does not directly bind to the promoter of IRF7 (Figure 5(f) and S5h). We thought that SURF6 might regulate the function of IRF7 through unknown transcription factors, warranting further investigation. To clarify whether SURF6 influences transcriptional activity of IRF7, we validated through dual-luciferase assays. We cloned the upstream promoter sequence of IRF7 for the dual-luciferase assay. When the plasmid of SURF6 was transfected into HEK293T cells, the activity of the WT promoter of SURF6 was significantly inhibited (Figure 5(g)). The results indicate that SURF6 suppresses the expression, nuclear translocation, and transcriptional activity of IRF7 in CRC cells. After establishing the regulation of IRF7 by SURF6, whether SURF6 impacts the control of the IFNβ promoter by IRF7 remains unknown. Dual-luciferase assays demonstrated that IRF7 activates the IFNβ promoter, which is suppressed by OE SURF6 (Figure 5(h)). This shows that as an upstream regulatory gene, SURF6 further inhibits the activation of the IFNβ promoter by suppressing the transcriptional activity of the transcription factor IRF7.

This study found that SURF6 suppresses the mRNA expression of IRF7 (Figure 5(b) and S5d), leading us to wonder whether SURF6 affects the RNA stability of IRF7. Using RT-qPCR on cells treated with actinomycin D (Act-D), we found that OE SURF6 does not affect the mRNA stability of IRF7 (Figure 5(i)). These results indicate that the regulation of IRF7 mRNA by SURF6 may not be related to RNA stability. To further clarify the relationship between miR3655/SURF6/IRF7/IFNβ, we transfected miR3655 mimics into KRAS G12D mutation cells and simultaneously overexpressed SURF6 to observe the protein levels of IRF7 and IFNβ (Figure 5j and S5i). We found that in KRAS G12D mutation cells, miR3655 mimics activated the expression of IRF7 and IFNβ, whereas SURF6 suppressed the expression of IRF7 and IFNβ. Additionally, the synergistic effect of miR3655 on IRF7 and IFNβ could be weakened by overexpressing SURF6. This further supports the regulatory relationship of miR3655 and SURF6 on IRF7/IFNβ. Moreover, through correlation analysis of clinical samples from Fujian Provincial Hospital, we found that miR3655 and SURF6 are negatively regulated (Figure 5(k)). And miR3655 positively regulated the IRF7/IFNβ (Figure 5(l,m)). SURF6 and IRF7/IFNβ are negatively regulated (Figure 5(n) and S5j). In summary, KRAS mutations suppress the expression of IRF7 and IFNβ. miR3655/SURF6 controls the activation of the IFNβ promoter by regulating the transcription factor IRF7. Specifically, the functions of SURF6 by influencing the transcriptional activity and nuclear translocation of IRF7, yet the specific mechanisms still require further study.

### Activation of IRF7/IFNβ inhibits intratumoral colonization of ETBF in CRC

**Figure 6.**
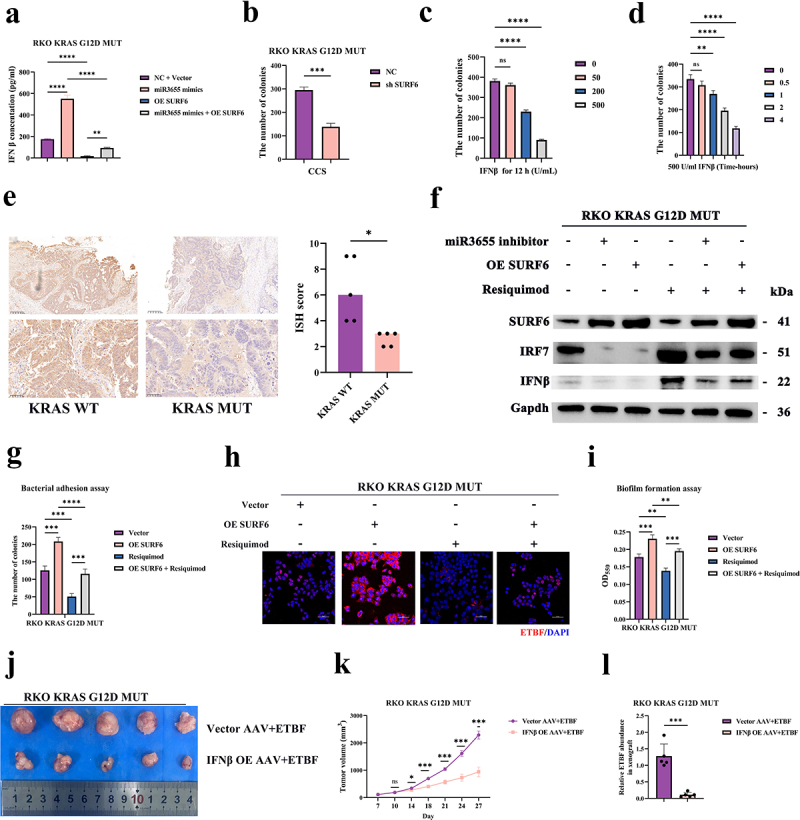
Activation of IRF7/IFNβ inhibits intratumoral colonization of ETBF in CRC.

After discovering the relationship between miR3655/SURF6/IRF7/IFNβ, we needed to further clarify whether the expression of miR3655 and SURF6 could affect the secretion of IFNβ by CRC. Initially, we examine the regulation of IFNβ secretion by miR-3655/SURF6 through a combination of ELISA and ultrafiltration tubes. We found that when the level of miR3655 was elevated, the secretion of IFNβ by CRC cells also increased (Figure 6(c) and S6a). OE SURF6 inhibited the secretion of IFNβ. The promotion of IFNβ secretion by miR3655 could be suppressed by OE SURF6. This supports not only the impact of miR3655/SURF6 on secretion of IFNβ in CRC but also further validating the regulatory relationship of miR3655/SURF6/IFNβ. After confirming the regulation of secretion of IFNβ in CRC by miR3655/SURF6, we hypothesized that IFNβ could have a direct inhibitory effect on ETBF independent of immune cells. We first added cell culture supernatant (CCS) from CRC cells cultured for 24 h to the culture medium containing ETBF. After 4 h, the amount of ETBF co-cultured with CCS under different conditions was observed on blood agar plates using the spread plate method. Surprisingly, compared with NC CCS, fewer ETBF survived when co-cultured with sh-SURF6 of CCS (Figure 6(b) and S6b). To determine whether IFNβ in CCS is responsible for killing ETBF, we treated ETBF directly with different concentrations of recombinant IFNβ protein. After 12 h of co-culture, the bacterial liquid was used for spread plate method. We found that 200 U/ml of IFNβ could significantly kill ETBF (Figure 6(c)). As the concentration of IFNβ increased, killing effect for ETBF became more significant, confirming the direct bactericidal activity of IFNβ with a concentration-dependent effect. Additionally, we treated ETBF with 500 U/ml of IFNβ for different times to investigate whether IFNβ has a time-dependent effect. We found that IFNβ could exert the direct bactericidal activity around 1 h (Figure 6(d)). With the extension of time, the bactericidal activity became increasingly significant. We further analyzed the effect of KRAS mutations on expression of IFNβ through IHC and confirmed that KRAS mutations inhibited expression of IFNβ (Figure 6(e)). In conclusion, KRAS mutations inhibited the direct bactericidal ability of IFNβ against ETBF.

(a) After treating CRC cells with miR3655 mimics and OE SURF6, the secretion level of IFNβ was measured using the ELISA method. (b) After knocking down SURF6, the number of surviving ETBF was observed using the dilution plating method after co-culturing CRC cell culture supernatant (CCS) with ETBF for 4 h. (c) After co-culturing with ETBF for 12 h and exogenously adding IFNβ protein, the killing effect of different concentrations of IFNβ on ETBF was observed using the dilution plating method. (d) The killing effect on ETBF at different times was observed using the dilution plating method after co-culturing with ETBF and exogenously adding 500 U/ml of IFNβ protein. (e) Immunohistochemical (IHC) of expression of IFNβ in CRC patients with KRAS MUT or KRAS WT (5 vs. 5). (f) After treating CRC cells with miR3655 inhibitors or OE SURF6, and adding the IRF7/IFNβ agonist Resiquimod, the expression levels of SURF6/IRF7/IFNβ were detected by WB. (g) The effect of CRC cells treated with OE SURF6 and addition of Resiquimod on the adhesive ability of ETBF. (h) FISH analysis of ETBF co-cultured with CRC cells that OE SURF6 and added Resiquimod, with each group repeated three times. (i) The effect on the biofilm formation capability of ETBF in CRC cells that OE SURF6 and added Resiquimod. (j) Images of xenograft tumors of nude mice from different experimental groups (5 mice per group). (k) Growth curves of xenograft tumors of nude mice across different experimental groups (5 mice per group). (l) qPCR analysis of the relative abundance of ETBF in xenograft tumors from different experimental groups (5 mice per group). (*p* > .05, ns; *p* < .05 *; *p* < .01 **; *p* < .001 ***; *p* < .0001 ****; Analysis performed using Student’s t-test, Wilcoxon rank-sum test, or Welch's ANOVA test for single or grouped analyses, respectively)

After confirming the direct bacterial-killing effect of IFNβ against ETBF, we hypothesized that by regulating the expression of IRF7 and IFNβ could affect the colonization of ETBF in CRC. Firstly, we used Resiquimod (R848; S28463), a TLR agonist, to enhance the expression of IRF7 and IFNβ (Figure 6(f) and S6c). In KRAS mutant cell lines, silencing miR3655 and OE SURF6 both reduced the expression of IRF7 and IFNβ (Figure 6f and S6c). The regulation of IRF7 and IFNβ by miR3655/SURF6 could be restored by Resiquimod, which further illustrates the regulatory relationship of miR3655/SURF6 with IRF7/IFNβ. The regulation of expression levels of IRF7 and IFNβ by SURF6 and Resiquimod was also reflected in the effect on adhesion capacity of ETBF. In KRAS MUT cells, OE SURF6 promoted the adhesion ability of ETBF, which was weakened by Resiquimod (Figure 6(g) and S6d). The inhibition of intratumoral colonization of ETBF by Resiquimod-activated IRF7/IFNβ was also confirmed by FISH (Figure 6(h) and S6e). We found that IFNβ not only affected the adhesion capability of ETBF but also significantly inhibited biofilms formation ability (Figure 6i and S6f). The aforementioned in vitro assays validated the involvement of IRF7/IFNβ in the KRAS/miR3655/SURF6-regulated colonization of ETBF in CRC. KRAS mutations promote the intratumoral colonization of ETBF in CRC by suppressing the secretion of IFNβ through the miR3655/SURF6/IRF7 axis.

**Figure 7.**
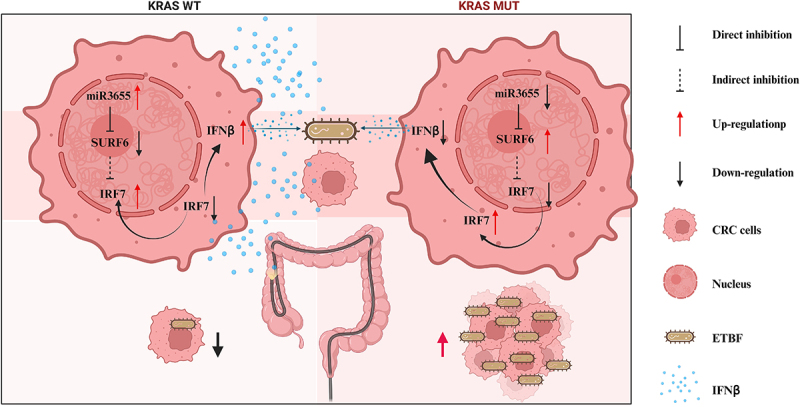
Molecular mechanisms of this study.

After establishing the in vitro effect of IFNβ, we used xenografted tumors of nude mice to verify whether IFNβ could inhibit intratumoral colonization of ETBF (Figure 6(j)). We found that the application of IFNβ OE AAV on KRAS G12D MUT xenografted tumors significantly inhibited tumor growth, volume, and weight (Figure 6(k) and S6G-S6H). qPCR also confirmed that IFNβ suppressed the abundance of ETBF in the tumor (Figure 6(l)). RT-qPCR validated the treatment efficacy of IFNβ OE AAV (Figure S6i). In summary, IFNβ can directly suppress the intratumoral colonization of ETBF. Both in vitro and in vivo assays have shown that KRAS mutations promote the intratumoral colonization of ETBF by suppressing the expression of IRF7/IFNβ (Figure 7).

The mechanistic diagram illustrates that KRAS mutations enhance intratumoral colonization of ETBF in CRC. Following KRAS mutations, the suppressed expression of miR3655 weakens the direct inhibitory effect on the target gene SURF6. In KRAS mutations CRC, the upregulated expression of SURF6 indirectly inhibits the transcription level and nuclear translocation of IRF7, thereby diminishing the capability of IRF7 to activate the promoter IFNβ. Consequently, KRAS mutations lead to a reduction in CRC-secreted IFNβ, promoting intratumoral colonization of ETBF in CRC.

## Discussion

For CRC, KRAS mutations and certain gut microbiota are risk factors, but it is unclear whether KRAS mutations can change the composition and diversity of the gut microbiota in CRC. To fill this gap, we used metagenomics to validate the feces of CRC patients with and without KRAS mutations. In our research, we unveiled that KRAS mutations facilitate proliferation of CRC by influencing the intratumoral colonization of ETBF for the first time. To our knowledge, miR3655 and SURF6, as new molecules for predicting the prognosis of CRC, are studied for the first time in CRC and the gut microbiome. Similarly, IFNβ has been demonstrated for the first time to have a direct bactericidal effect on ETBF. Considering that interventions based on the tumor microbiome have already shown great therapeutic potential,^54^ this study provides new mechanistic insights.

The differences in microbial clustering and diversity changes observed in this metagenomics reveal the potential impact of genetic alterations on the microbiome landscape. KRAS mutations drive different microbial spectra, which may influence progression of disease and treatment efficacy. Additionally, regulating the microbiome within the TME is becoming increasingly valued in tumor therapy, making ETBF and potential biomarkers as therapeutic targets in KRAS-mutant CRC. This study confirmed that regardless of the KRAS status, there are individual differences in the gut microbiome of all patients. This variability highlights the personalization of the microbiome and supports the necessity of individualized approaches in developing microbiome-based diagnostic or therapeutic methods.^55^ Furthermore, the complex analysis network and LEfSe further clarified the differential microbial spectrum associated with KRAS mutations. The increase in the abundance of bacteroides in KRAS mutant CRC patients is consistent with previous studies,^35^ providing another layer of evidence for the interaction between certain bacteria and CRC genetic subtypes. As a classic carcinogenic bacterium in the bacteroides, ETBF is fundamentally important in the initiation and progression of CRC.^56^ Researchers can study the interaction between ETBF (including Gammaproteobacteria and Pseudomonadota) and KRAS mutations in CRC. Unlike this study, previous research used 16S ribosomal RNA gene sequencing on tumor tissues.^35^ The similar results between CRC tumor tissue and fecal sequencing may be reasonably explained by the unique anatomical characteristics of CRC, allowing free exchange between intratumoral microorganisms and gut microbiota, which requires further large-sample omics validation. In summary, the status of KRAS might be a key factor regulating the balance of the gut microbiota. In addition, the causality between KRAS mutations and the gut microbiome warrants further investigation.

TME screens certain intratumoral microorganisms, allowing them to thrive. After gut microbiota colonize within CRC, intratumoral microbes also need to adapt to the TME to survive. We validated in CRC patients that the KRAS mutation positively correlates with the abundance of ETBF in feces, aligning with our metagenomic findings. Similarly, the high load of FN in CRC is associated with KRAS mutations,^57^ again highlighting the potential link between gut microbiota and the genetic characteristics. Recent studies have also shown the correlation between ETBF and molecular features of CRC, suggesting that an ETBF-rich microbiome may be related to specific CRC subtypes.^58^ This finding echoes our view that KRAS mutations and the microbiome may both contribute to the molecular pathogenesis of CRC. Moreover, KRAS mutations may create a favorable TME for gut microbiota, inducing carcinogenic bacteria to colonize within the tumor. Research is limited on how tumor cells manipulate gut microbiota to co-construct the TME. Studies have confirmed that bile acid metabolic disorders form a unique gut microbiome.^26^ Dysregulated bile acid metabolism positively regulates secretory immunoglobulin A, enhancing the biofilm formation of ETBF, ultimately promoting CRC.^26^ Unlike this study, that research focused on the impact of metabolism on the ETBF and did not investigate the manipulation of ETBF by tumor. Another study found that the TME provided by normal intestinal epithelial cells does not allow FN to colonize, unlike CRC cells.^30^ High expression of ANGPTL4 promotes glycolytic activity in CRC cells, which is essential for colonization of FN in CRC. That study is also from a metabolic perspective, different from the molecular subtypes/immune molecule perspective in our study (by secreting IFNβ). Our study confirmed that compared to KRAS WT, KRAS mutation promotes intratumoral colonization of ETBF. However, further research is necessary. We did not deeply explore whether the gut microbiota can lead to KRAS mutations, which is an intriguing research direction. Investigating the interaction between other gut microorganisms and KRAS mutations is also crucial for future research.

miRNAs are valuable for the diagnosis and prognosis prediction of CRC patients.^59,60^ Gut microbiota can regulate processes of CRC such as proliferation, drug resistance, and metastasis through miRNAs.^61–63^ Some studies have shown that miRNAs can enter bacteria, specifically regulating gene transcription and affect bacterial growth.^41^ Additionally, KRAS mutations can facilitate tumorigenesis by impacting several steps, including processing of miRNA.^64^ It appears that tumors and the gut microbiota have formed a close connection through miRNAs. Our research systematically describes the impact of miR3655 against the backdrop of KRAS mutations and infection of ETBF on CRC, revealing innovative value as a new biomarker and potential therapeutic target. These findings provide new perspectives and potential strategies for miRNA-based diagnosis and personalized treatment in CRC. Consistent with our research, miR3655 has also shown tumor-suppressive effects in the prognostic prediction model of pancreatic cancer.^65^ We found that miR3655 not only affects the proliferation of CRC cells but also suppresses intratumoral colonization of ETBF in CRC. This new finding paves the way for future treatments related to ETBF infection and KRAS mutations. Additionally, SURF6, as a nucleolar protein involved in ribosome biogenesis, has not been researched in cancer and gut microbiota.^66^ In line with miR3655, SURF6, as a new biomarker predicting CRC, is also involved in regulating intratumoral colonization of ETBF in CRC. Notably, the promotion of proliferation of CRC by SURF6 requires the involvement of ETBF. While our study provides new insights in these fields, future in-depth research is still required to elucidate more comprehensive biological effects in interactions between miR3655 and SURF6. Moreover, it is particularly important to validate these findings in a broader patients and study potential clinical applications in the TME.

Our study discovered that SURF6 can inhibit the expression of the TLR signaling pathway and downstream effector factors IRF7 and IFNβ for the first time. The TLR signaling pathway, an important part of the immune system, plays a significant role in host defense against pathogen invasion and suppressing tumors.^67,68^ SURF6 promotes the survival of carcinogenic microbes by interfering with the immune defense mechanisms of the host. Notably, our research provides evidence of SURF6 as a potential immunoregulatory factor in CRC. This finding not only enriches our understanding of the immunological microenvironment regulation in CRC but also lays the groundwork for developing potential therapeutic strategies targeting microorganisms. Further research should explore the specific molecular bridging mechanisms behind miR3655/SURF6 regulation of the IRF7/IFNβ axis. Research on IFNs has spanned a century, and as new discoveries emerge, the complexity of the IFN-related network continues to increase. IFNβ acts as a tumor suppressor in various cancers. IFNβ promotes tumor cell dormancy in melanoma patients,^69^ and exerts anti-tumor activity in CRC when combined with immunotherapy.^70^ Our study reveals the direct bactericidal effect of IFNβ on ETBF for the first time. Previous research identified parts of the surface in IFNβ molecule that have cationic and amphipathic properties, which are classic features of antimicrobial peptides.^53^ IFNβ can directly kill staphylococcus aureus,^53^ which also supports the findings of our study. However, this study did not investigate whether IFNβ kills ETBF through a function similar to antimicrobial peptides. Future research should investigate the potential applications of IRF7/IFNβ in KRAS mutant CRC, as well as the regulation of tumor-associated microbial ecology through the miR3655/SURF6 axis, providing insights into personalized treatment strategies.

Through a multi-layered assay approach, this study unveiled a new mechanism by which KRAS mutations promote intratumoral colonization of ETBF in CRC. For the first time, the critical role of the miR3655/SURF6/IRF7/IFNβ axis in the KRAS mutation-regulated intratumoral colonization of ETBF in CRC has been clearly demonstrated. This reveals a completely new regulatory network, offering a fresh perspective on the complex interplay between KRAS mutations, CRC, and microbiota. Future research should aim to expand these findings, establish causality, and elucidate the potential mechanisms driving these associations. While these data provide novel insights into the relationship between KRAS mutations and the microbiome, further large-scale clinical studies and in-depth mechanistic research are required to confirm this relevance and offer a more detailed biological explanation.

## Supplementary Material

Supplemental Material

## Data Availability

Data for this study may be requested from the corresponding author where appropriate.
