# Supplementary material for: KRAS mutations promote the intratumoral colonization of enterotoxigenic bacteroides fragilis in colorectal cancer through the regulation of the miRNA3655/SURF6/IRF7/IFNβ axis
Source: Gut Microbes. 2024 Nov 10;16(1):2423043. doi: 10.1080/19490976.2024.2423043 (PMC11556274; doi:10.1080/19490976.2024.2423043)
Supplement: Supplemental Material [file KGMI_A_2423043_SM9679.docx]

**Figure S1. KRAS mutations alter the composition and diversity of the gut microbiota in CRC**


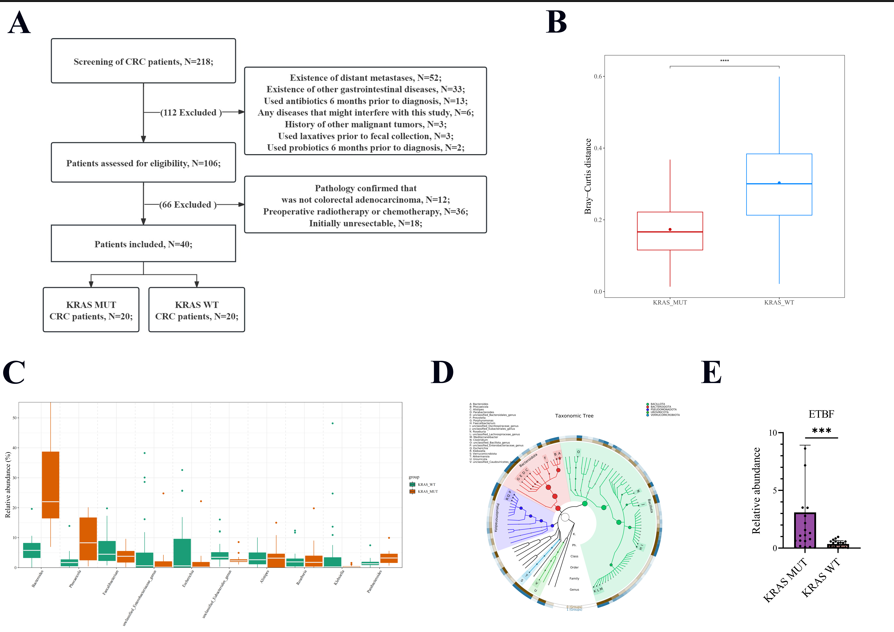


(A) A flowchart for the prospective collection of tumor tissues and fecal samples from CRC patients (20 vs. 20). (B) Box plot of the distances between gut microbiota from CRC patients with KRAS WT versus KRAS MUT (20 vs. 20; p<0.001). (C) Relative abundance box plot. Dominant genus with high abundance is selected for quartile calculations. (D) Hierarchical annotation GraPhlAn visualization. Based on the abundance analysis of the genus, dominant genus is selected and combined with genus and functional hierarchy information for visualization and annotation. (E) Differences in the abundance of ETBF between groups were analyzed. (P > 0.05, ns. nonsignificant; P < 0.05 *; P < 0.01 **; P < 0.001 ***; P < 0.0001 ****; Analysis performed using Student’s t-test, Wilcoxon rank-sum test, or Welch ANOVA test for single or grouped analyses, respectively)

**Figure S2. KRAS mutations promote the intratumoral colonization of ETBF in CRC**


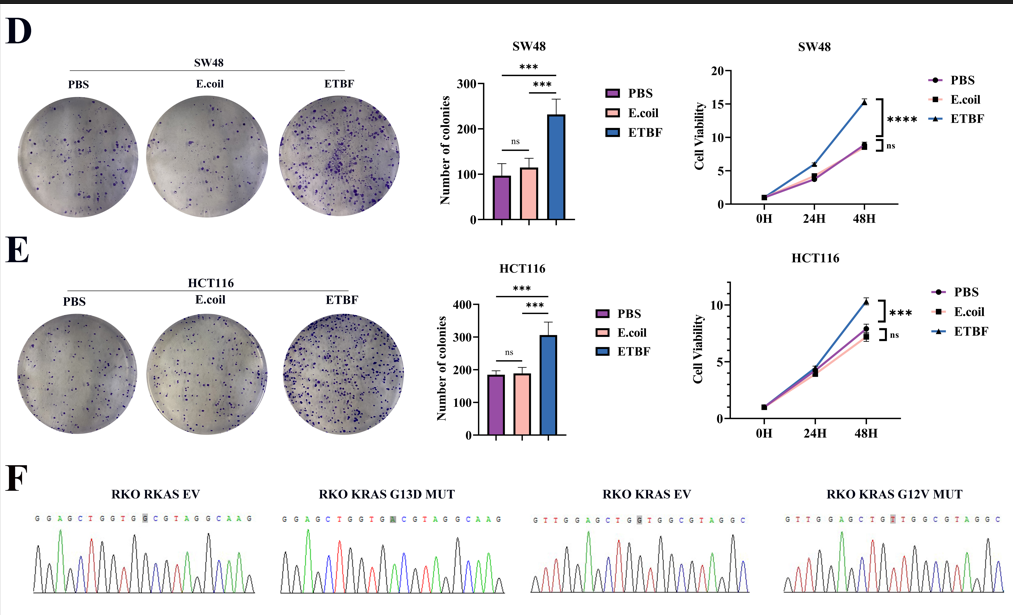


(A) The CCK-8 assay was used to evaluate the effects of the KRAS G12D MUT on the proliferation of CRC cells. (B) Colony formation assays and quantitative analyses were conducted on different groups of CRC cells. (C) Sanger sequencing was utilized to detect the presence of the KRAS G12D mutation in RKO and SW48 cell lines. (D) Colony formation assays and CCK-8 assays were performed on SW48 cells with or without infection of ETBF. (E) Colony formation assays and CCK-8 assays were carried out on HCT116 cells with or without infection of ETBF. (F) Sanger sequencing identified the presence of KRAS G12V mutation and KRAS G13D MUT in the RKO cell line. (G) Growth curves of xenograft tumors of nude mice across different experimental groups (5 mice per group) were charted, with tumor volumes assessed at specified intervals. (H) Weights of xenograft tumors were measured at the end of the experiment in different experimental groups (5 mice per group). (P > 0.05, ns; P < 0.05 *; P < 0.01 **; P < 0.001 ***; P < 0.0001 ****; Analysis performed using Student’s t-test, Wilcoxon rank-sum test, or Welch ANOVA test for single or grouped analyses, respectively)

**Figure S3. KRAS mutations promote intratumoral colonization of ETBF in CRC by inhibiting miR3655**

**
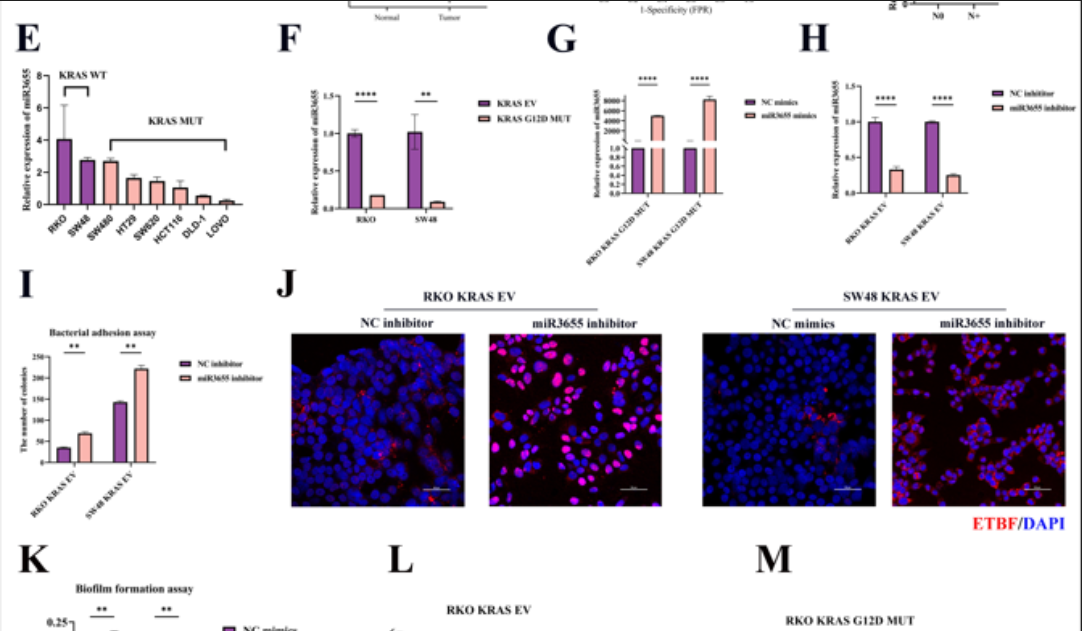
**

(A) Key miRNAs related to KRAS mutations and colitis in CRC were identified using the GEO database. GEO: Gene Expression Omnibus. (B) The expression of miR3655 between tumor tissues and normal tissues in the TCGA database. TCGA: The Cancer Genome Atlas. (C) The capability of miR3655 to diagnose CRC in the TCGA database was analyzed using Receiver Operating Characteristic (ROC) curves. (D) The expression of miR3655 among CRC patients with different N stages (n=80 CRC patients). (E) The expression of miR3655 in CRC cells with KRAS WT and KRAS MUT were detected by RT-qPCR. (F) The expression of miR3655 in CRC cells with KRAS EV and KRAS G12D MUT were detected using RT-qPCR. (G and H) RT-qPCR was used to detect the expression of miR3655 in CRC cells after treated with miR3655 mimics and inhibitors. (I) The effect of miR3655 inhibitor on the adhesive ability of ETBF in CRC cells. (J) FISH analysis of ETBF co-cultured with KRAS EV CRC cells treated with miR3655 inhibitors, with each group repeated three times. (K) The impact of miR3655 inhibitor on the biofilm formation capability of ETBF in CRC cells. (L) The CCK-8 assay to assess the effect of miR3655 inhibitor on the proliferation of CRC cells. (M) The volume of xenograft tumors on day 28 in different experimental groups of nude mice (5 mice per group). (P > 0.05, ns; P < 0.05 *; P < 0.01 **; P < 0.001 ***; P < 0.0001 ****; Analysis performed using Student’s t-test, Wilcoxon rank-sum test, or Welch ANOVA test for single or grouped analyses, respectively)

**Figure S4. miR3655 inhibits intratumoral colonization of ETBF in CRC by targeting the KRAS mutation-associated molecule SURF6**


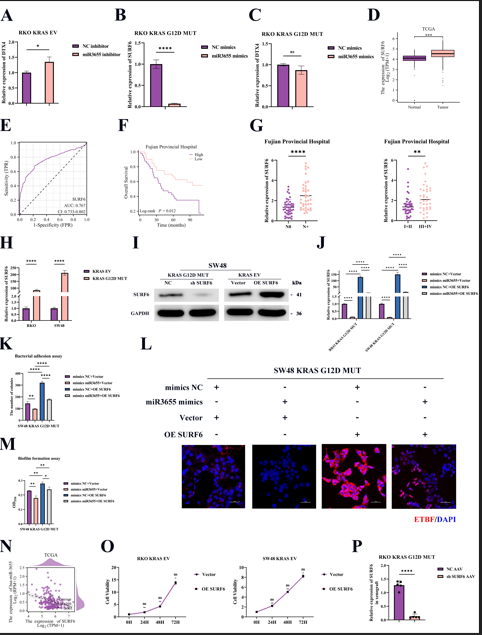


(A) RT-qPCR was used to detect the expression level of DTX4 in CRC cells treated with miR3655 inhibitors. (B) RT-qPCR was used to detect the expression level of SURF6 in CRC cells treated with miR3655 mimics. (C) RT-qPCR was used to detect the expression level of DTX4 in CRC cells treated with miR3655 mimics. (D) The expression of SURF6 between tumor tissues and normal tissues in the TCGA database. (E) Using ROC curve analysis to assess the ability of SURF6 to diagnose CRC in TCGA. (F) KM survival analysis on the OS of CRC patients with high or low expression of SURF6 (n=80 CRC patients). (G) The expression of SURF6 in CRC patients with different N stages and TNM stages (n=80 CRC patients). (H) RT-qPCR was used to detect the expression of SURF6 in CRC cells with KRAS EV or KRAS G12D MUT. (I) WB was used to measure the protein level of SURF6 in CRC cells after silencing and OE SURF6. (J) RT-qPCR was used to detect the impact of OE SURF6 on the expression of SURF6 in CRC cells treated with miR3655 mimics. (K) The impact of OE SURF6 on the adhesive ability of ETBF in CRC cells after miR3655 mimics treatment. (L) After treated with miR3655 mimics and subsequently OE SURF6, these cells were co-cultured with ETBF. FISH analysis was performed, with each group repeated three times. (M) The impact of OE SURF6 on the biofilm formation capability of ETBF in CRC cells after miR3655 mimics. (N) Pearson analysis of the correlation between miR3655 and SURF6 in TCGA. (O) The CCK-8 assay to detect the effect of OE SURF6 on the proliferation of CRC cells. (P) RT-qPCR to measure the expression of SURF6 after intratumoral injection of AAV in xenograft tumors of nude mice. (P > 0.05, ns; P < 0.05 *; P < 0.01 **; P < 0.001 ***; P < 0.0001 ****; Analysis performed using Student’s t-test, Wilcoxon rank-sum test, or Welch ANOVA test for single or grouped analyses, respectively)

**Figure S5. SURF6 suppresses the Toll-like receptor signaling pathway and the expression of IRF7/IFNβ in CRC**

**
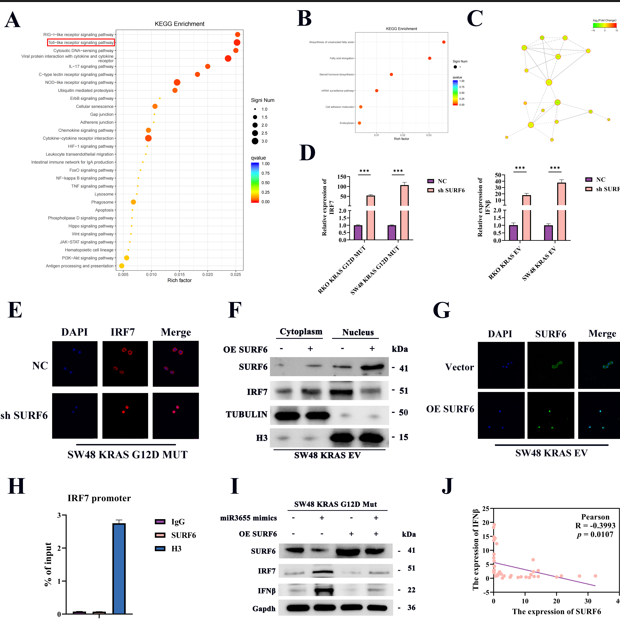
**

(A-B) Pathway analysis revealed the main signaling pathways that are inhibited and activated in CRC cells after OE SURF6. (C) Protein-protein interaction network diagram. (D) RT-qPCR analysis revealed the expression of IRF7 and IFNβ in CRC cell lines after sh SURF6. (E) Following the knockdown of SURF6 in CRC cells, IF (red) showed the expression of IRF7; DAPI (blue) indicated the nucleus. (F) Detected the expression of SURF6 and IRF7 in the subcellular compartments of CRC cells after OE SURF6 by WB. (G) IF (green) showed the expression of SURF6 in CRC cells after OE SURF6; DAPI (blue) displayed the nucleus. (H) ChIP-PCR analysis to detect whether there is binding between SURF6 and the IRF7 promoter. (I) Detected the expression of IRF7 and IFNβ after OE SURF6 in CRC cells treated with miR3655 mimics by WB. (J) Pearson analysis explored the correlation between SURF6 and IFNβ of CRC patients in Fujian Provincial Hospital. (P > 0.05, ns; P < 0.05 *; P < 0.01 **; P < 0.001 ***; P < 0.0001 ****; Analysis performed using Student’s t-test, Wilcoxon rank-sum test, or Welch ANOVA test for single or grouped analyses, respectively)

**Figure S6. Activation of IRF7/IFNβ inhibits intratumoral colonization of ETBF in CRC**

**
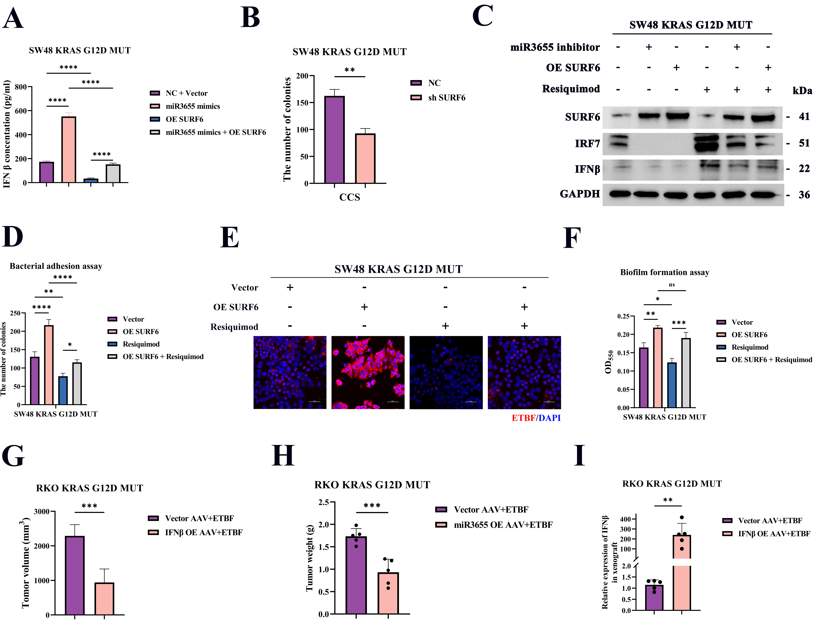
**

(A) After treating CRC cells with miR3655 mimics and subsequently OE SURF6, the secretion level of IFNβ was measured using the ELISA method. (B) After knocking down SURF6, the CCS of CRC cells was co-cultured with ETBF for 4 hours. And the number of surviving ETBF was observed using the dilution plating method. (C) After treating CRC cells with miR3655 inhibitors or OE SURF6, and adding the IRF7/IFNβ agonist Resiquimod, the expression levels of SURF6/IRF7/IFNβ were detected by WB. (D) The effect on the adhesive ability of ETBF in CRC cells after OE SURF6 and adding Resiquimod. (E) FISH analysis of ETBF co-cultured with CRC cells that OE SURF6 and added Resiquimod, with each group repeated three times. (F) The impact on the biofilm formation capability of ETBF in CRC cells after OE SURF6 and addition of Resiquimod. (G) The volume of xenograft tumors on day 28 in different experimental groups of nude mice (5 mice per group). (H) The weight of xenograft tumors was measured at the end of the experiment in different experimental groups of nude mice (5 mice per group). (I) RT-qPCR was used to measure the expression level of SURF6 after intratumoral injection of AAV in xenograft tumors of nude mice. (P > 0.05, ns; P < 0.05 *; P < 0.01 **; P < 0.001 ***; P < 0.0001 ****; Analysis performed using Student’s t-test, Wilcoxon rank-sum test, or Welch ANOVA test for single or grouped analyses, respectively)

**Table S1. Clinic-pathological characteristics of CRC patients**

| Case Number | Gender | Age | TNM stage | KRAS staus |
| --- | --- | --- | --- | --- |
| 1 | Female | 52 | I | G12D |
| 2 | Female | 58 | III | G12V |
| 3 | Female | 65 | II | G12D |
| 4 | Female | 58 | I | G12D |
| 5 | Male | 69 | II | G12C |
| 6 | Male | 71 | II | G12D |
| 7 | Female | 50 | II | G12V |
| 8 | Male | 56 | II | G12D |
| 9 | Male | 55 | II | G13D |
| 10 | Male | 59 | III | G12V |
| 11 | Male | 75 | II | G13D |
| 12 | Male | 73 | II | G12D |
| 13 | Male | 52 | II | G12V |
| 14 | Female | 54 | II | G13D |
| 15 | Male | 64 | III | G12D |
| 16 | Female | 69 | II | G12C |
| 17 | Male | 60 | II | G12D |
| 18 | Male | 73 | II | G12D |
| 19 | Female | 58 | II | G13D |
| 20 | Female | 49 | III | G12V |
| 21 | Female | 55 | I | WT |
| 22 | Female | 49 | II | WT |
| 23 | Female | 66 | III | WT |
| 24 | Male | 67 | II | WT |
| 25 | Male | 73 | II | WT |
| 26 | Male | 62 | I | WT |
| 27 | Male | 70 | II | WT |
| 28 | Male | 71 | II | WT |
| 29 | Male | 64 | II | WT |
| 30 | Female | 69 | III | WT |
| 31 | Male | 60 | II | WT |
| 32 | Male | 49 | II | WT |
| 33 | Male | 59 | I | WT |
| 34 | Male | 65 | II | WT |
| 35 | Female | 62 | II | WT |
| 36 | Female | 55 | III | WT |
| 37 | Male | 46 | II | WT |
| 38 | Female | 59 | II | WT |
| 39 | Female | 57 | II | WT |
| 40 | Male | 68 | I | WT |

WT: wild-type.

**Table S2. Baseline characteristics of the including CRC patients.**

| Variables^*^ | KRAS WT | KRAS MUT | P^†^ |
| --- | --- | --- | --- |
| N | 20 | 20 |  |
| Age (years) |  |  |  |
| ≤65/>65 | 12/8 | 13/7 | 0.744 |
| Gender |  |  |  |
| Female/Male | 8/12 | 9/11 | 0.749 |
| ECOG PS |  |  |  |
| 0-1/2-3 | 16/4 | 16/4 | 1.000 |
| TNM |  |  |  |
| I-II/III | 17/3 | 16/4 | 1.000 |
| CEA at diagnosis, ng/mL |  |  |  |
| ≤5/>5 | 16/4 | 15/5 | 1.000 |

*: The dietary habits of these patients were consistent.

†: Pearson’s *x*^2^ test or continuity correction test was used to analyze the basic characteristics.

CEA: carcinoembryonic antigen; ECOG PS: Eastern Cooperative Oncology Group performance status.

**Table S3. Primer sequence of PCR**

| Gene | Forward Primer (5'-3') | Reverse Primer (5'-3') |
| --- | --- | --- |
| **Human** |  |  |
| miR3655 | TAATGCTTGTCGCTGCGGTGTTG | Universal Reverse Primer |
| U6 | CTCGCTTCGGCAGCACA | Universal Reverse Primer |
| GAPDH | GGTGTGAACCATGAGAAGTATGA | GAGTCCTTCCACGATACCAAAG |
| SURF6 | CTCTCTACTCGCCAAGGACG | GAGCCTTGAGTTTTGCCAGC |
| DTX4 | TCTGTATGGAACGCCTCACG | GGTCTTGCAGGTTGGACACT |
| IRF7 | ATGGGCAAGTGCAAGGTGTA | GATGGTATAGCGTGGGGAGC |
| IFNβ | GCTGGAATGAGACTATTGTTGAGA | GCCTTCAGGTAATGCAGAATCC |
| **Bacteria** |  |  |
| ETBF | GAGCCGAAGACGGTGTATGTGATTTGT | TGCTCAGCGCCCAGTATATGACCTAGT |
| PGT | ATCCCCAAAGCACCTGGTTT | AGAGGCCAAGATAGTCCTGGTAA |
| 16 S | CGTCAGCTCGTGTCGTGAG | CGTCGTCCCCACCTTCC |

**Table S4. Antibody information**

| Antibody | Dilution | Application | Cat number | Manufacturer |
| --- | --- | --- | --- | --- |
| SURF6 | 1:1000 | WB/IF | sc-515439 | Santa Cruz |
| IRF7 | 1:2000 | WB/IF | DF7503 | Affinity |
| IFNβ | 1:1000 | WB | DF6471 | Affinity |
| TUBULIN | 1:10000 | WB | 10094-1-AP | Proteintech |
| H3 | 1:5000 | WB/CHIP | ab1791 | abcam |
| GAPDH | 1:10000 | WB | ab181602 | abcam |
| Goat Anti-Mouse lgG H&L (Alexa Fluor® 488) | 1:200 | IF | ab150113 | abcam |
| Goat Anti-Rabbit lgG H&L (Alexa Fluor® 647) | 1:200 | IF | ab150079 | abcam |
| Goat Anti-Rabbit IgG (H+L) HRP | 1:5000 | WB | S0001 | Affinity |
| Goat Anti-Mouse IgG (H+L) HRP | 1:5000 | WB | S0002 | Affinity |

**Table S5. miRNA、Lentiviral and AAV construct sequence**

|  | Forward sequence (5′ to 3′) |
| --- | --- |
| miR3655 mimics | GCUUGUCGCUGCGGUGUUGCU |
| miR3655 inhibitor | AGCAACACCGCAGCGACAAGC |
| KRAS G12D | ATGACTGAATATAAACTTGTGGTAGTTGGAGCTGATGGCGTAGGCAAGAGTGCCTTGACGATACAGCTAATTCAGAATCATTTTGTGGACGAATATGATCCAACAATAGAGGATTCCTACAGGAAGCAA |
| KRAS G12V | GCCTGCTGAAAATGACTGAATATAAACTTGTGGTAGTTGGAGCTGGTGTCGTAGGCAAGAGTGCCTTGACGATACAGCTAATTCAGAATCATTTTGTGGACGAATATGATCCAACAATAGAG |
| KRAS G13D | GCCTGCTGAAAATGACTGAATATAAACTTGTGGTAGTTGGAGCTGGTGACGTAGGCAAGAGTGCCTTGACGATACAGCTAATTCAGAATCATTTTGTGGACGAATATGATCCAACAATAGAG |
| sh SURF6 | GCTGATCTTCAATAAGGTGGA |
| OE SURF6 | NCBI Reference Sequence: NM_006753.6 |
| miR3655 OE AVV | CGCTGCGAGGTCAATTTCTTTGCGCTCGGGGTTGGTCGGAGGGAAAAACAGGAAGCGGAAAGGCTGCGAACGCAAAGCAGTGTGGGTTGATTCTGAGGTGCACTGTGGGAAAGAGCTTGTCGCTGCGGTGTTGCTGTTGGAGACTCGATTGTTGGTGACAGCGAAAGAACGATAACAAAATGCCGGAGCGAGATAGTAAGGCTCAGG |
| IFNβ OE AAV | NCBI Reference Sequence: NM_002176.4 |
